# PBPK Modeling Approach to Predict the Behavior of Drugs Cleared by Kidney in Pregnant Subjects and Fetus

**DOI:** 10.1208/s12248-021-00603-y

**Published:** 2021-06-24

**Authors:** Ke Xu Szeto, Maxime Le Merdy, Benjamin Dupont, Michael B. Bolger, Viera Lukacova

**Affiliations:** 1 Simulations Plus, Inc., 42505 10th Street West, Lancaster, California, 93534 USA; 2 PhinC Development, 36 Rue Victor Basch, 91300 Massy, France

**Keywords:** *in silico*, PBPK, pregnancy, renal clearance

## Abstract

The purpose of this study was to develop a physiologically based pharmacokinetic (PBPK) model predicting the pharmacokinetics (PK) of different compounds in pregnant subjects. This model considers the differences in tissue sizes, blood flow rates, enzyme expression levels, glomerular filtration rates, plasma protein binding, and other factors affected during pregnancy in both the maternal and fetal models. The PBPKPlus™ module in GastroPlus^®^ was used to model the PK of cefuroxime and cefazolin. For both compounds, the model was first validated against PK data in healthy non-pregnant volunteers and then applied to predict pregnant groups PK. The model accurately described the PK in both non-pregnant and pregnant groups and explained well differences in the plasma concentration due to pregnancy. The fetal plasma and amniotic fluid concentrations were also predicted reasonably well at different stages of pregnancy. This work describes the use of a PBPK approach for drug development and demonstrates the ability to predict differences in PK in pregnant subjects and fetal exposure for compounds excreted renally. The prediction for pregnant groups is also improved when the model is calibrated with postpartum or non-pregnant female group if such data are available.

**Supplementary Information:**

The online version contains supplementary material available at 10.1208/s12248-021-00603-y.

## INTRODUCTION

The impact of medication used during pregnancy on both maternal and fetal health is a growing public health concern with the steady increase of medication intake over the last three decades. The average number of medications (both prescription and over the counter) taken by pregnant women increased by 68% between 1977 and 2007 with now an average of 4.2 active pharmaceutical ingredients (APIs) used during pregnancy [1].

These APIs are required to ensure the health of the pregnant woman and fetus [2]. Drug safety during pregnancy and lactation must be demonstrated by sponsors during drug development. However, clinical development programs for a new API rarely include pregnant women, with the exception of drugs designed for obstetric use, due to ethical reasons [3,4]. For this reason, non-clinical studies (in animals and *in vitro* cell or tissue experiments) are the most prominent sources of information during the drug development period to assess drug safety in this population [5,6]. Despite being informative, these non-clinical methods remain imperfect due to major interspecies differences or scalability issues of *in vitro* systems. A historic example of a public health crisis, such as thalidomide [7], illustrates this discrepancy between non-clinical approach and real life experience. Besides the safety concerns, it is noticeable that the doses administered to pregnant women are extrapolated from observed data in non-pregnant female populations, without consideration of the profound physiological changes occurring during pregnancy [8–10]. Modification of body composition and impact on the cardiovascular, digestive, and renal systems can significantly alter the pharmacokinetics of an API. Because of these safety concerns and lack of specific dose adjustments, both the pregnant woman and the fetus are exposed to an increased risk of incorrect pharmacotherapy with sub- or supra-therapeutic drug exposure leading to toxic effects in the woman and/or fetus. In recent years, multiple research efforts focusing on pharmacokinetics (PK) and pharmacodynamics (PD) of APIs administered to the pregnant population were performed. The use of physiologically based pharmacokinetic (PBPK) modeling has emerged as a comprehensive tool to investigate and predict clinical trials involving a pregnant population [11].

PBPK models integrate both API physicochemical information and physiological parameters that describe in a mechanistic way the succession of anatomical, physiological, and physical events involved in API absorption, distribution, metabolism, and excretion processes. API excretion usually occurs through renal mechanisms either as an unchanged drug or after metabolic processes. In the kidney, a combination of passive filtration and active secretion or reabsorption contributes to the renal clearance of the unchanged drug. Plasma free fraction, glomerular filtration rate (GFR), and kidney size are important physiological parameters influencing the renal excretion and are known to change during pregnancy [8,10]. Therefore, systemic clearance and excretion of API and/or its metabolites will change.

PBPK models that are first validated in a non-pregnant population can be used to predict the PK exposure of an API during pregnancy, by accounting for the physiological differences between pregnant and non-pregnant women. The first PBPK models dedicated to pregnancy predictions were developed in the 1990s for rodents [12], to enhance extrapolation form preclinical studies, based on their abilities to account for interspecies differences. In subsequent years, these models served as a basis for further improvements in prediction of human health risk, while other models were also developed to extrapolate animal pregnancy models to human. However, the lack of human data, particularly for fetal exposure, resulted in a high degree of uncertainty about the applicability of predictions from these models. Over the last decade, several PBPK models dedicated to human predictions have been published [13–16].

The purpose of this research was to develop and validate a PBPK model predicting the maternal and fetal PK of various APIs administered to women during different stages of pregnancy. These account for the modifications in tissue sizes, blood flow rates, enzyme expression levels, GFR, plasma protein binding, and other factors in both maternal and fetal physiologies at all stages of pregnancy. The developed model also predicts the PK in several fetal and surrounding tissues such as cord blood and amniotic fluid. This paper describes the developed model and the physiological changes throughout pregnancy. As an initial step, this model was validated for drugs being cleared by the kidney using clinical data for two antibiotics commonly used during pregnancy: cefuroxime (CFX) and cefazolin (CZ).

## MATERIAL AND METHODS

### Physiology

**Fig. 1:**
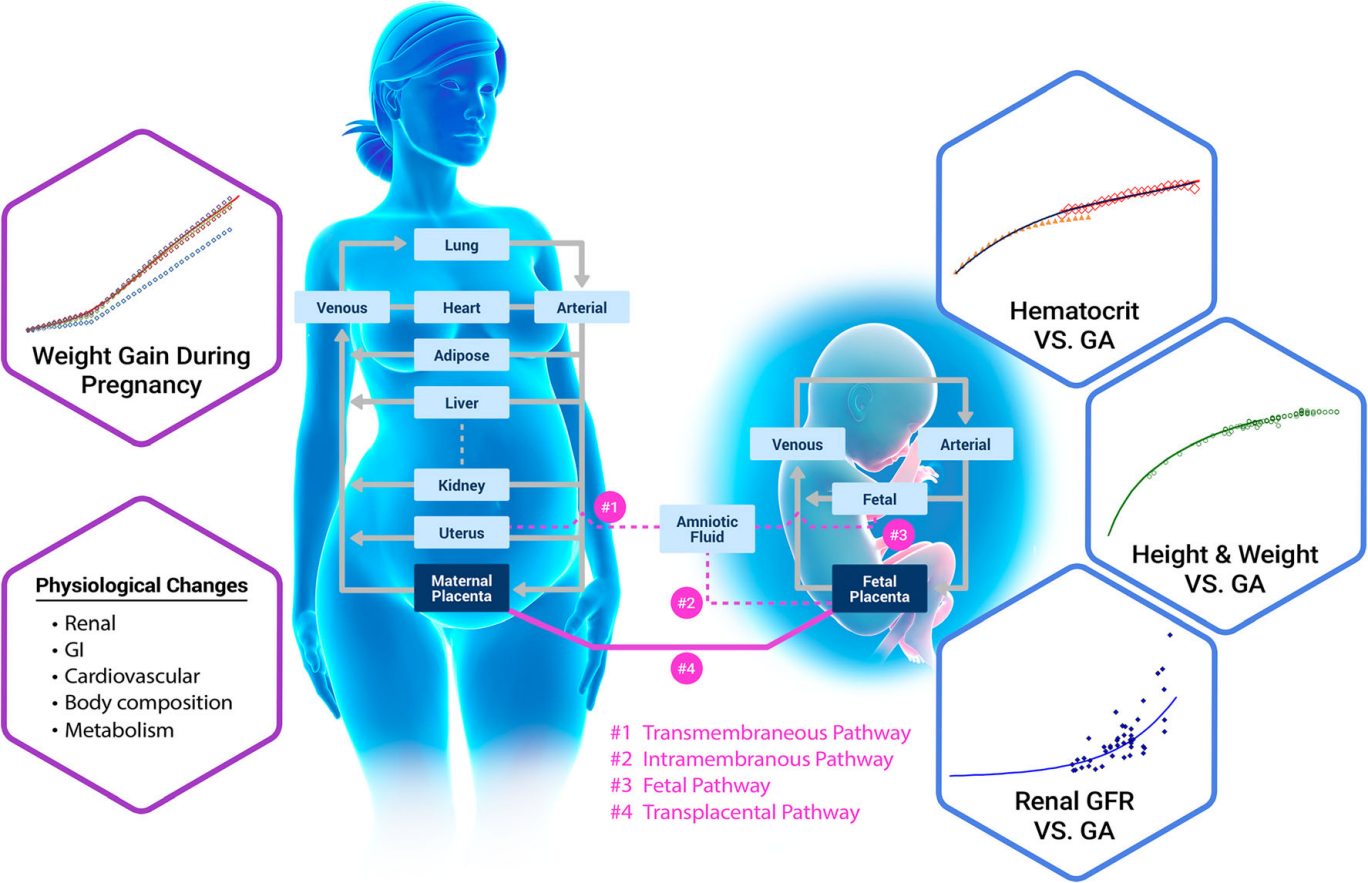
PBPK model representation for pregnant woman. Weight gain and physiological changes during pregnancy are based on pre-pregnancy BW and GA. The fetus PBPK model is composed of four compartments: venous blood, arterial blood, fetal tissue, and fetal placenta. The amniotic fluid compartment is also created, and its volume depends on GA

Pregnancy incurs a series of changes in the maternal body [8–10,15,17–19] such as weight gain, changes in enzyme expression levels, enlargement of certain tissues such as uterus, placenta, brain, and kidney, increased amount of plasma volume, gastrointestinal (GI) changes (increased stomach transit time), *etc.* The volume of fetal tissue and fetal blood will also increase with gestational age (GA). The duration of normal pregnancy to term is 38 to 42 weeks. The physiological changes incorporated in the model as a function of maternal age, pre-pregnancy weight, GA, and tissue changes are briefly described in this section and all model specific equations are presented in supplementary materials 1 and 2. Figure 1 gives a broad overview of some of the changes occurring in both the maternal and fetal physiology.

#### Maternal Weight Gain

One of the major changes in the maternal physiology is weight gain. The total body weight for the maternal subject is the sum of pre-pregnancy body weight and the weight gain during pregnancy. The pre-pregnancy weight is used to calculate the normal subject tissue weights. For tissues that do not change with pregnancy such as heart, spleen, liver, and lung, the tissue weight remains unchanged during pregnancy. For tissues which do change during pregnancy, such as skin, adipose, kidney, placenta, and uterus, the percent of increase in tissue size is calculated and added to the pre-pregnancy tissue weight. Carmichael *et al.* [20] have published weight gain percentiles for different body mass index (BMI) groups. The underweight, normal weight, and overweight subjects have consistent measurements throughout the pregnancy, while the obese group showed significantly lower weight gain compared to the other groups. Similar results were also presented in another study [21]. In the present model, the average weight gain from all underweight, normal weight, and overweight subjects is implemented and the resulting plots from the corresponding equations are presented in supplementary material 2.

#### Other Maternal Changes

The maternal changes in brain volume, plasma volume, hematocrit, plasma protein concentration, GFR, uterus weight, and kidney volume are based on previously published equations [8,10]. The skin volume, adipose volume, cardiac output, and other relevant changes were calculated by Population Estimates for Age Related (PEAR) physiology based on the current body weight (pregnancy weight + weight gain) at different gestation ages in pregnancy by the equations for healthy subjects. Transporters (influx and efflux) expression levels are scaled based on the organ volume.

#### Fetal Changes

The data for fetal GFR, hematocrit, liver size, blood volume, and plasma protein levels were limited or not available, especially at early gestation ages. Therefore, they were either extrapolated from data for term/preterm infants or previously published equations (see supplemental material 2).

##### Fetal Weight and Height

The fetal weight and height at early pregnancy (before 12 weeks) were extrapolated from the preterm infant and fetus measurements.

##### Hematocrit and Glomerular Filtration Rate

Hematocrit *versus* GA is based on the Dallmann equation [10] and available observed data for infants [22–24]. GFR *versus* GA was extrapolated from data for infants (data presented in supplementary material 1). In the same graph, the continuous changes of hematocrit and GFR ontogeny are also shown after birth.

##### Amniotic Fluid Volume

The volume of amniotic fluid changes with GA. The equation that calculated this volume was adopted from Dallmann *et al.* [10]. The dynamics of the amniotic fluid transport mechanisms is discussed in detail in the “Mechanisms” section.

##### Other Fetal Changes

The other measurements, such liver size, blood volume, and plasma protein levels, were either extrapolated from current infant equations or based on the available data for early pregnancy.

### Mechanisms

There are three important transport pathways to balance the fluid and solute between the fetal tissue and the environment around it: intramembranous, fetal, and transmembranous pathways [25,26]. The intramembranous pathway accounts for the transfer between the amniotic fluid and the fetal blood within the placenta and membranes; transmembranous pathway accounts for transfer between amniotic fluid and maternal blood within the wall of the uterus. The fetal pathways define the exchanges between the fetal organs and amniotic fluid mediated by swallowing by the fetus, oral, nasal, and respiratory tract secretion, and urine from the fetus (Fig. 1).

The passive transmembranous pathway (*K*_*trans*_) is based on the free concentration difference between the amniotic fluid and the uterus tissue. In early gestation ages, since the other pathways are not developed, the transmembranous pathway is the major pathway governing the transport between fetal tissue and the maternal environment.

The homeostasis of amniotic fluid from the active pathways (both fetal and intramembranous) is described by the following equation accounting for both intramembranous and fetal pathways:

$$ Urinary\ rate+{K}_{sec}={K}_{sw}+{K}_{intram} $$

where *K*_*sec*_ is the secretion rate from the fetal tissue, *K*_*sw*_ is the fetal swallowing rate, and *K*_*intram*_ is the active part of intramembranous pathway. From this equation, the active inflow and outflow rates from and into the amniotic fluid compartment are balanced.

The passive and active rate constants (*Urinary rate*, *K*_*sec*_, *K*_*sw*_, *K*_*intram*_, *K*_*trans*_) for the water and solute movements are not well established for human. However, some measurements are available for ovine fetus [25,27,28]. In those publications, the authors have provided the values of these four active rates either from other literature sources or their own model estimates. The sums of these rates were calculated to be 1.26 and 1.51 L/day [17,25]. In the current model, for each parameter, the average of the two values in these two publications was calculated, while maintaining the total balance of the fluid. The resulting sum of fluid transfer is calculated as 1.46 L/day (urinary rate + lung secretion: *K*_*sec*_) and the passive rate was estimated from [23]. These rates are then normalized by the fetal birth weight (3.32 kg) and multiplied by the fetal body weight to calculate the rate constants at different GA.

The urine starts to form around 9–12 weeks and so are the other fetal pathways. Therefore, all the above rate constants related to the intramembranous and fetal pathways ( *Urinary rate*, *K*_*sec*_, *K*_*sw*_, *K*_*intram*_) are set to be 0 when GA is less than 9 weeks old. For the values of the individual rate constants, please refer to supplemental material 1.

### Model Validation

Two case studies were identified to validate the PBPK model ability to predict maternal and fetal PK at different stages of pregnancy for drugs mainly eliminated by the kidney: CFX and CZ. These compounds are only eliminated by the kidney in unchanged form. In both cases, sufficient *in vitro* and *in vivo* information was available in literature to develop and validate the PBPK models. Baseline PBPK models for non-pregnant healthy adults were developed using GastroPlus (version 9.8 Simulation Plus Inc., Lancaster, CA, USA). The PBPK physiology was then changed to pregnant and the clinical trials involving pregnant women were predicted. In all simulations, maternal plasma concentrations (Cp) as well as available fetal concentrations (*e.g.*, fetal venous return, placenta, amniotic fluid) were simulated and compared with observed data from clinical studies. The compound properties, model settings, and clinical studies used for the model development, validation, and extrapolation to the pregnant population for both compounds are summarized in Supplementary material 3.

#### CFX PBPK Model

The baseline PBPK model in healthy non-pregnant subjects was developed with all tissues defined as perfusion limited except for the kidney that is defined as permeability limited. The tissue/plasma partition coefficients (Kp) were calculated using the default methods for each tissue type: Lukacova method for the perfusion limited tissues [29] and the Poulin and Theil extracellular method for the permeability limited tissues [30]. Renal filtration was estimated as fraction unbound in plasma (fup) × GFR. The tubular secretion was modeled via organic anion transporter 3 (OAT3) influx transporter on the basolateral membrane and multidrug resistance protein 4 (MRP4) efflux transporter on the apical membrane of the kidney [31]. The K_m_ value for OAT3 transporter was extracted from literature [31]. Due to the lack of current literature data for the affinity of CFX to MRP4, the K_m_ value was considered similar to OAT3.

#### CZ PBPK Model

The baseline PBPK model was developed with all tissues defined as perfusion limited except for the kidney that is defined as permeability limited. Kps were calculated using the default methods: Lukacova method for the perfusion limited tissues and the Poulin and Theil extracellular method for the permeability limited tissues [29,30]. Renal filtration was estimated as fup ∗ GFR. The tubular secretion was modeled via OAT3 influx transporter on the basolateral membrane and MRP4 efflux transporter on the apical membrane of the kidney. Experimental K_m_ value for hOAT3 is not available and was approximated by experimental K_i_ value (117 μM) from *in vitro* measurement of ^3^H-estrone sulfate uptake inhibition by cefazolin in HEK-292 cells [32]. For MRP4, the K_m_ value was set to the experimental K_m_ (80.9 μM) measured in membrane vesicles [33].

## RESULTS

### Cefuroxime

**Fig. 2:**
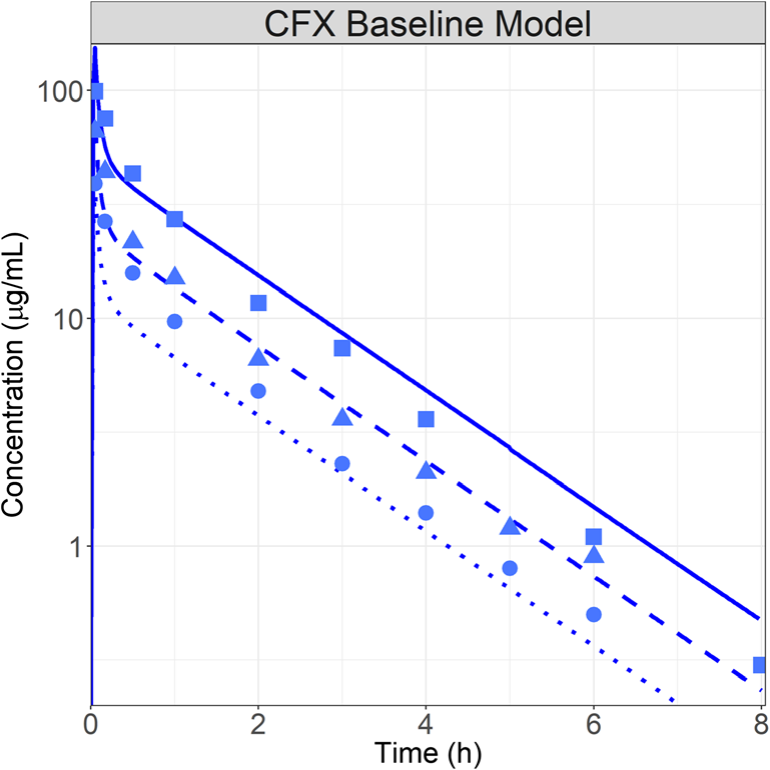
CFX baseline PBPK model development: simulated (lines) and observed (points) (34) profiles after administration of CFX (●, dotted line: 0.25; ▲, dashed line: 0.5; ■, solid line: 1 g) IV infusion in healthy fasted volunteers (30-year-old, 70 kg male subjects)

CFX is a cephalosporin antibiotic clinically used to treat several infections during pregnancy. This drug is either administered intravenously (IV) or intramuscularly (IM). Although the clearance is not affected by the dose, the volume of distribution tends to increase as the dose increases [34]. CFX is completely cleared via the kidneys as unchanged drug through glomerular filtration and tubular secretion. The baseline PBPK model was developed using clinical data following administration of 0.25, 0.5, and 1 g IV infusion for 3 min [34]. The V_max_ values for both transporters were fitted simultaneously across all three dose levels [34]. Overall, the model described the plasma concentration time profiles very well [34] (Fig. 2). The total urinary secretion presented in literature (around 95 to 99% of the dose secreted in urine) is also captured accurately (data presented in supplementary material 4).

**Fig. 3:**
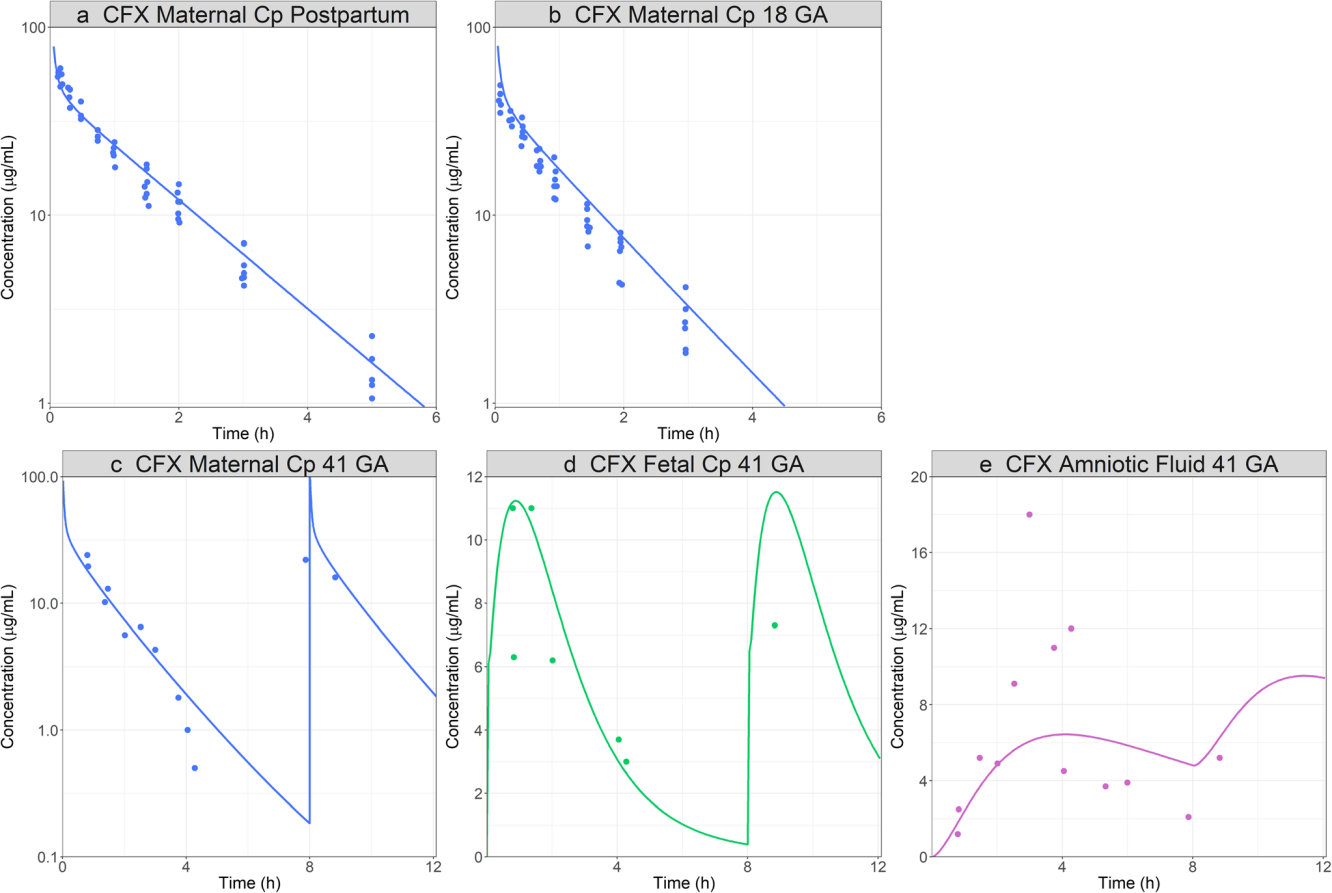
CFX pregnant PBPK model development and validation: simulated and observed (35) PK profiles after administration of 0.75 g IV bolus CFX to a 30-year-old, 61.7 kg female. **a** Postpartum Cp time profile and percent of dose secreted in urine. **b** Cp time profile and percent of dose secreted in urine at 25 GA. **c** Cp time at 41 GA. **d** Fetal Cp time profile at 41 GA. **e** Amniotic fluid concentration profile at 41 GA

The model was used to simulate CFX PK in pregnant women at 18 and 41 weeks of gestation, as well as postpartum after their menstrual cycles were normal and they were not breastfeeding [35]. For the non-pregnant (postpartum) stage, although plasma concentration profile was well predicted, the urinary secretion was overpredicted (simulated > 95% and observed 76%). Therefore, the MRP4 transporter V_max_ was reduced by five-fold to match the urinary data (Fig. 3a). The adjusted model based on postpartum data was used to simulate maternal Cp time profile in pregnant population at 18 and 41 GA (Fig. 3b and c) as well as the reported fetal plasma and amniotic fluid concentration during delivery (41 GA). The plasma concentrations were predicted accurately for both groups. The predicted total % urinary excretion of ~ 85% within first 8 h after the second administration is also in line with the observed data (84%) [35]. The increase in urinary excretion is due to the well-known increase in GFR that occurs in pregnant women [8]. At 41 weeks of gestation, the initial predictions for fetal plasma and amniotic fluid concentration time course showed a higher C_max_ and shorter T_max_ compared to the observed data. Therefore, placenta was changed to a permeability limited tissue model. The permeability parameter (permeability-surface area product, PStc) was fitted to capture the fetal plasma concentration profile (final PStc for maternal placenta (15.9 mL/s) and fetal placenta (32,400 mL/s) at 41 GA). The fetal kidney clearance was set to fetal fup ∗ fetal GFR. The final model with the adjusted PStc parameters correctly captured the fetal plasma as well as amniotic fluid concentrations following the administration of 0.75 g in a 41 GA weeks pregnant woman (Fig. 3d and e).

**Fig. 4:**
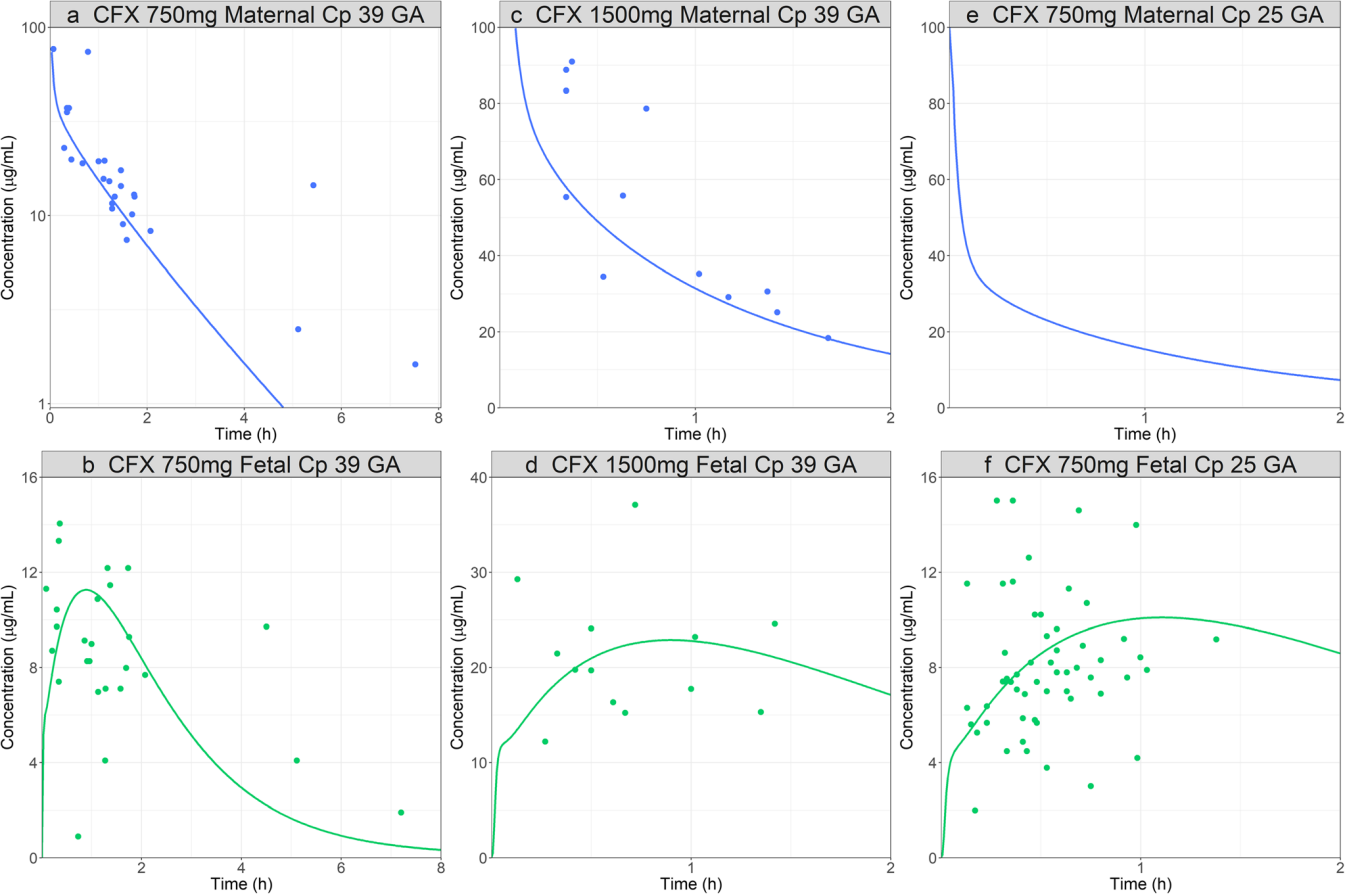
CFX pregnant PBPK model validation: simulated and observed (36,37) maternal (top) and fetal (bottom) PK profiles after administration of CFX. **a**, **b** Cp time profiles following 0.75 g IV bolus of CFX in a 30-year-old, 81 kg female pregnant subject, 39 weeks GA. **c**, **d** Cp time profiles following 1.5 g IV bolus of CFX in a 30-year-old, 84 kg female pregnant subject, 39 weeks GA. **e**, **f** Cp time profiles following 0.75 g IV bolus of CFX in a 30-year-old, 84 kg female pregnant subject, 25 weeks GA

The model was validated by predicting maternal and fetal Cp *vs.* time profiles following the CFX administration at different doses (0.75 g and 1.5 g) and at different pregnancy stages (25 and 39 GA weeks) [36,37]. The maternal and fetal placental PStc were scaled for each study based on their volumes. Overall, the model predicted both maternal and fetal CFX PK well (Fig. 4a to f).

In all studies, observed fetal tissue concentrations presented a significant intersubject variability. As the studies designs only allowed for one cord blood and/or amniotic fluid sample per subject, the overall concentration time course is also not accessible for individual subjects. To insure the validated PBPK model could describe the observed variability, a population simulation was performed to describe the maternal and fetal Cp as well as the amniotic fluid concentrations following the administration of 0.75 g IV bolus CFX. The population setting (range of GA, weight, BMI, weight gain, and age of the subjects) used in the simulation matched the information provided in the original paper (7 subjects in total) [35]. GastroPlus population simulator generates pregnant virtual subjects based on a bivariate distribution of height and weight relevant for the selected age of a non-pregnant subject and then applies the weight gain for the defined GA. The default variability (CV%) for individual physiological parameters is based on information obtained from literature. Values within range of 10–20% are assumed for parameters where intersubject variability was not found in literature. The CV% for the V_max_ values of both transporters are assumed to be 100% based on their protein abundance levels in human kidney [38]. Final simulation results are presented in supplementary material 4. Overall, the model could predict relatively well the observed variability in both maternal and fetal tissues.

### Cefazolin

**Fig. 5:**
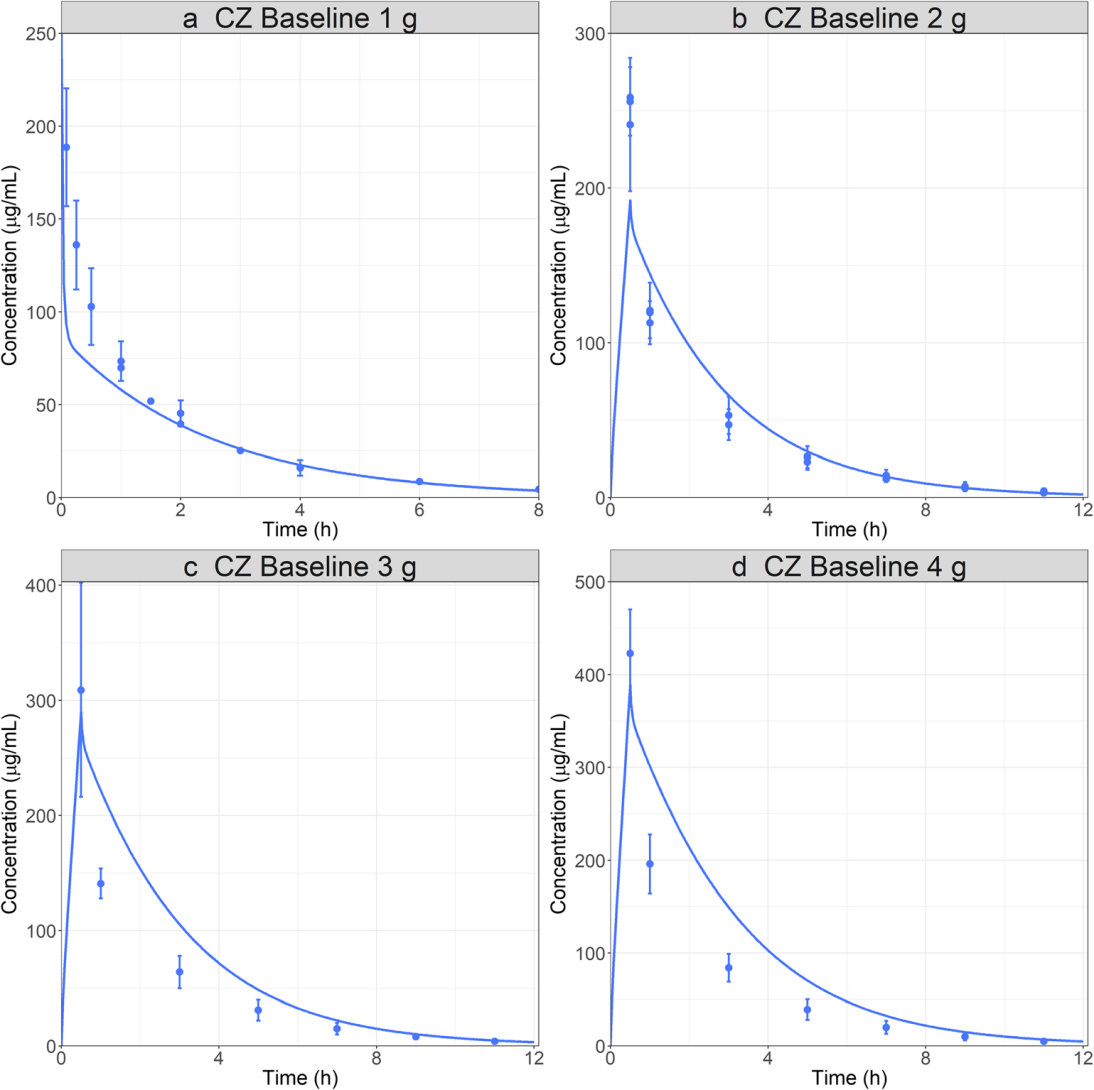
CZ baseline PBPK model development: simulated and observed (41–44) PK profiles following administration of CZ: **a** 1 g IV bolus for a 32-year-old, 79 kg male subject; **b** 2 g; **c** 3 g; **d** 4 g IV infusion 30 min for a 28-year-old, 67 kg male subject

CZ is an antibiotic clinically used for antibacterial prophylaxis during several surgical procedures in pregnant women. CZ is completely cleared via the kidneys as unchanged drug through glomerular filtration and tubular secretion mediated by OATs 1/3 and MRP4 [33,39,40]. The PBPK model was developed and validated using clinical data for 1, 2, 3, and 4 g IV bolus or infusion (0.5 h) administration [41–44]. The V_max_ values for both transporters were fitted to match the PK data for 1 g dose. The data for 2, 3, and 4 g doses were subsequently used for external model validation. Overall, the model describes the Cp time profiles adequately for all datasets (Fig. 5). For all doses, the first hours following the end of infusion are not well described by the model. Based on parameter sensitivity analysis, this might due to nonlinear CZ plasma protein binding [45]. When the total plasma concentration is lower than 100 μg/mL, the fup is expected to be less than 10%. As the total plasma concentration increases above 100 μg/mL, the fup increases and therefore, the filtration portion of the kidney clearance (fup ∗ GFR) increases in proportion. Simulations performed with a higher fup better described the observed data for the first 3 h but then underpredicted the terminal concentrations. The saturable plasma protein binding is not currently included in GastroPlus; therefore, an fup of 9% was used as an average compromise value and resulted in reasonable simulations for of plasma concentrations for all four dose groups.

**Fig. 6:**
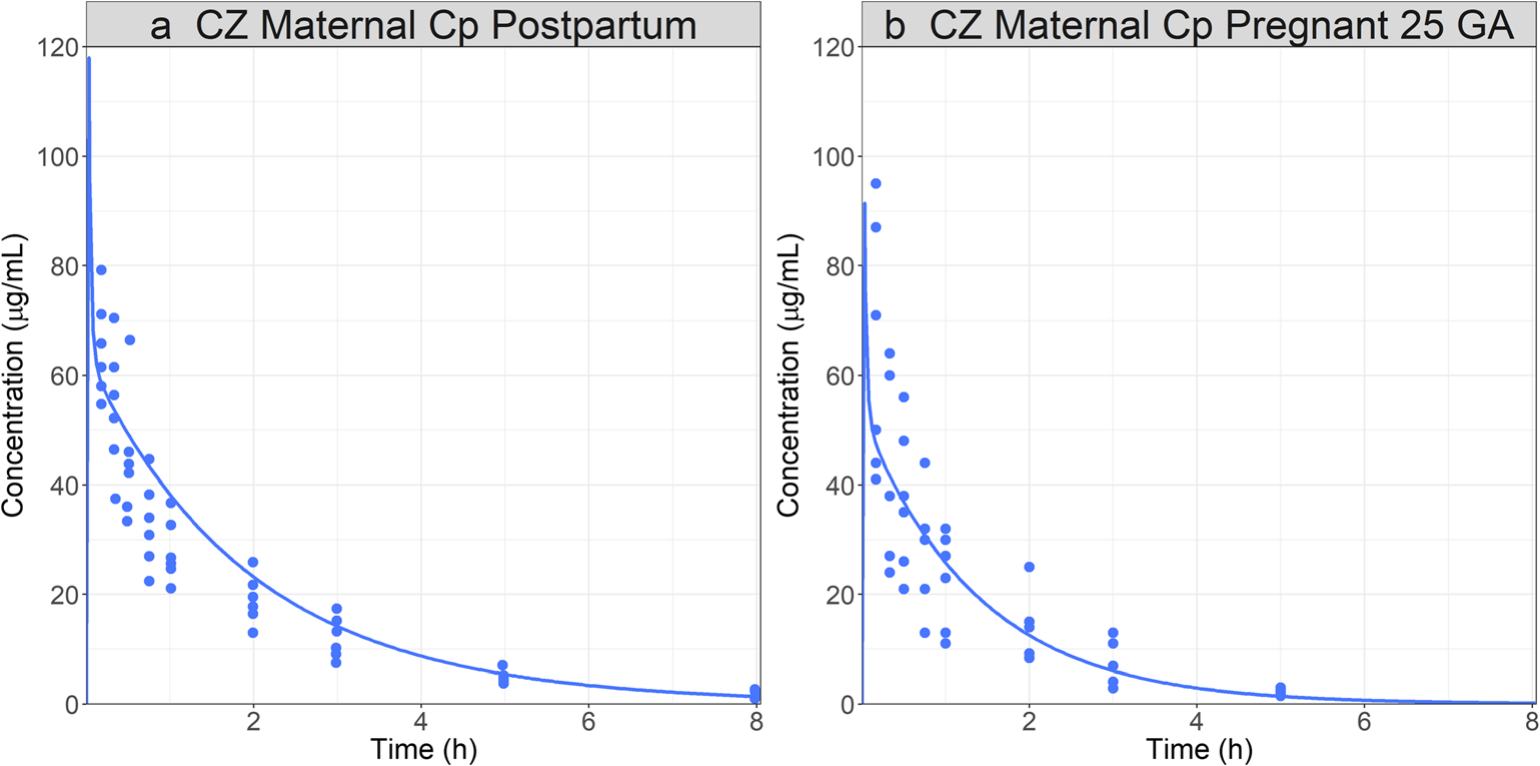
CZ pregnant PBPK model development and validation: simulated and observed (46) Cp time profiles after administration of CZ to a 25-year-old, 62 kg female subject: **a** postpartum stage; **b** at 25 weeks GA with a weight gain of 6 kg during pregnancy

The model was used to simulate CZ PK after administration 0.5 g IV bolus dose in pregnant women at 25 weeks of gestation and postpartum after their menstrual cycles were normal and they were not breastfeeding [46]. The model accurately predicted CZ PK postpartum (Fig. 6a) and was subsequently used to simulate the PK profile in the same subjects during pregnancy (Fig. 6b). By changing the physiological parameters from non-pregnant to pregnant, the model accurately predicted the PK for CZ in a pregnant population at a dose of 0.5 mg at 25 GA weeks.

**Fig. 7:**
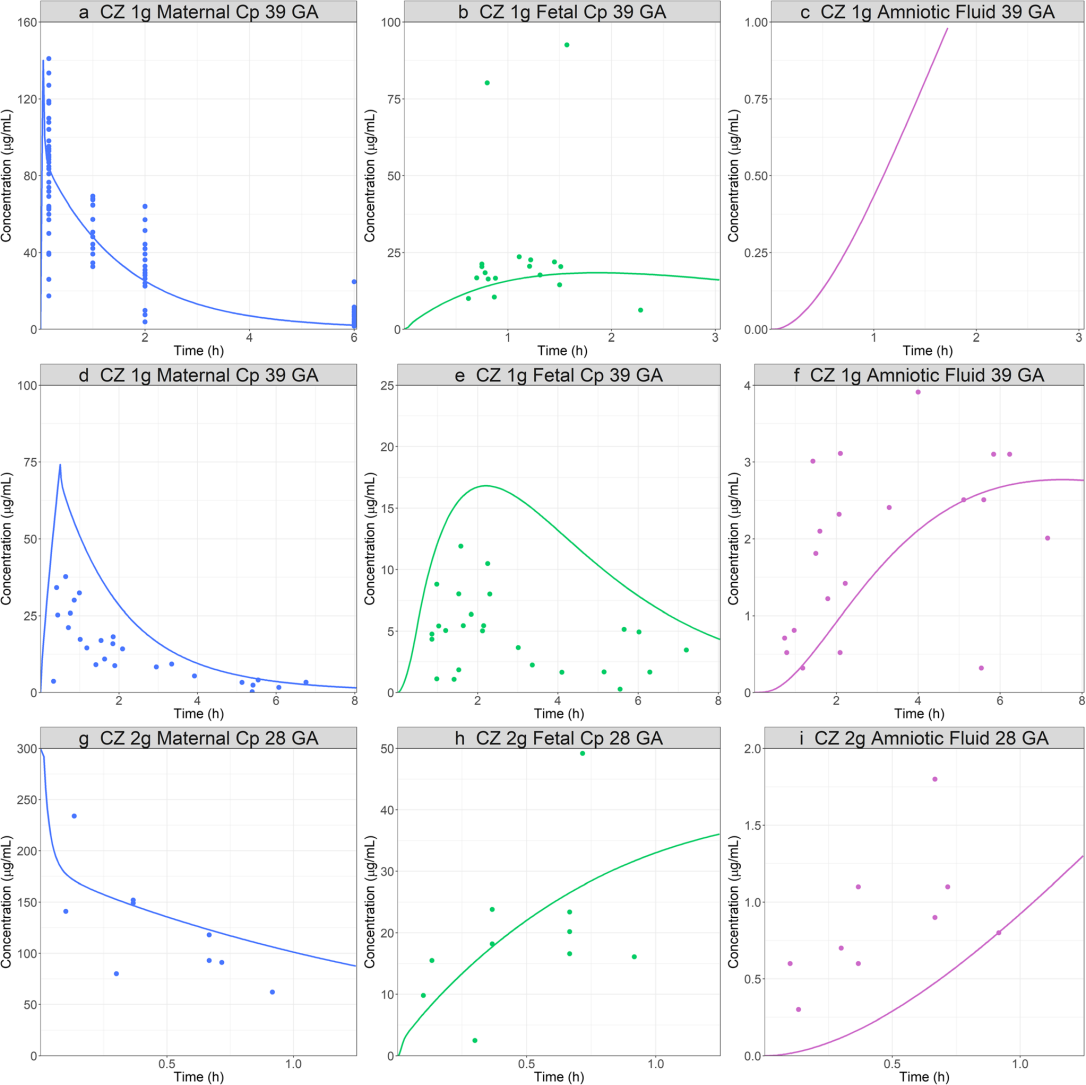
CZ pregnant PBPK model validation: simulated and observed (47–49) PK profiles after administration of CZ. **a**, **b**, **c** Maternal CP, fetal CP, and amniotic fluid concentration time profiles following 1 g IV bolus of CZ in 33-year-old, 79 kg female pregnant subjects, 39 weeks GA. **d**, **e**, **f** Maternal Cp, fetal Cp, and amniotic fluid concentration time profiles following 1 g IV infusion of CZ (infusion time was not reported in the publication and 30 min infusion was assumed) in 31-year-old, 91 kg (body weight for a typical 31-year-old female at 39 weeks of gestation) female pregnant subjects, 39 weeks GA. **g**, **h**, **i** Maternal Cp, fetal Cp, and amniotic fluid concentration time profiles following 2 g IV bolus of CZ in a 30-year-old, 73 kg female pregnant subject, 28 weeks GA. The fetal kidney filtration was set to fetal fup ∗ fetal GFR

The model was subsequently used to predict maternal and fetal PK of CZ following the IV administration of CZ at different doses and pregnancy stages [47–49]. Similar to CFX, the initial simulations for fetal plasma and amniotic fluid concentration time course presented a higher C_max_ and shorter T_max_ compared to the observed data. Therefore, placenta was again changed to permeability limited tissue model. A specific PStc value of 1 mL/s/mL tissue was fitted to match the fetal plasma concentration profile from one of the clinical studies [47]. This specific PStc value provided also estimates for PStc in kidney tissue and was tested in the model for healthy volunteers. The incorporation of this mechanism improved the prediction for urinary secretion (final Cp time profiles are presented in Figs. 5 and 6; urinary data are presented in supplementary material 4). The same placenta tissue settings were subsequently used in all remaining simulations. In all cases, the fetal kidney clearance was set to fetal fup ∗ fetal GFR. Overall, the final model adequately predicted the maternal and fetal plasma as well as amniotic fluid concentrations following the IV administration of 1 or 2 g in pregnant women at 28 or 39 weeks of gestation (Fig. 7). Some of the mispredictions may be explained by incomplete information in the published studies. Fiore Mitchell *et al.* [48] study did not provide the body weights of the study subjects and the default body weight for a typical 31-year-old female at 39 GA (91 kg) was used in the simulation. An incorrect assumption on the body weight could explain the difference between the observed and simulated data. Because this study included only five patients and there is large intersubject variability in other studies [47], it did not seem necessary to further refine the model at this stage. The shapes of the maternal and fetal Cp time profiles as well as their ratios are predicted well, indicating the overprediction of the fetal plasma concentration is linked to the maternal misprediction. In both studies that reported amniotic fluid concentrations [48,49], the model underpredicts these concentrations. Fetal kidney tubular secretion is not implemented in the model as no information could be found about the transporter expressions in the fetal kidney at this time. This missing mechanism for fetal kidney tubular secretion might contribute to the underprediction of the amniotic fluid concentrations. In addition, the patients in the Brown study [49] were presenting signs of hydrops and/or signs of an excess in bilirubin in this fluid. The current PBPK model considers a healthy pregnancy, and the disease state could affect the physiology and contribute to mispredictions in amniotic fluid concentration. In both cases, the fetal kidney clearance parameter can be modified to better describe the data (data not shown).

## DISCUSSION

This study describes a newly developed human pregnancy PBPK model. It consists of a whole body PBPK model on the maternal side and a simplified PBPK model on the fetal side. The model was applied to simulate PK for two compounds which are exclusively cleared by renal excretion, including active secretion by transporters. The maternal PK was predicted accurately for all studies. The fetal PK was also reasonably described after adjustments of placenta tissue compound specific permeabilities.

The fetal model is composed of four compartments that includes a fetal tissue, fetal blood, amniotic fluid, and fetal placenta (portion of the placenta tissue facing the fetal blood circulation) compartments. As the pregnancy evolves, these compartments and their connections are changing based on the physiological information collected from literature [8–10,18,19]. Although the fetal compartment size changes during pregnancy, it does not subdivide into individual fetal organs which is a limitation of the current model structure. Inclusion of all the important organs in the fetus, especially at the later stages of the pregnancy when the organ functions become more developed, will be necessary to predict the local concentrations in the fetal organs in order to evaluate drug efficacy and safety as well as control toxicity. However, validation of such a complex model would be challenging as the data for concentrations in individual tissues is not available. Indeed, recent studies developing a complete PBPK model for the fetus were only used for simulations purposes but the results were not compared to observed fetal concentrations [50,51]. A recent review of PBPK models used for pregnant women demonstrated that in a vast majority of cases, only the maternal Cp time profile prediction was investigated, justifying a simplified model structure. Only a few cases were also interested in fetal blood cord exposure [52]. Therefore, as an initial step, a simplified fetal PBPK model was created as data were available to perform multiple validation cases study. This current fetal model structure matches the most complex fetal PBPK models focusing on prediction of maternal and fetal PK at different pregnancy stages [15,17,53,54]. Recent publications presented pregnancy PBPK models that include additional maternal tissues compartments: breast, endometrium, and myometrium [55,56]. However, these organs are not connected to the placenta and therefore should have no or limited impact on APIs transplacental maternal to fetal transfer. In addition, these models have other limitations as the exchange between the amniotic fluid and the fetus is not considered. Pregnancy PBPK model for renally eliminated drugs was also enhanced recently to account for ethnic differences by including specific details for Chinese pregnant populations [57]. Ongoing research regarding placental tissue structure and the consequences on fetal exposure will support enhancement of PBPK model for pregnancy applications [58,59]. The accurate predictions of both fetal Cp and amniotic fluid concentrations demonstrate the great potential of such models to impact drug development for a safety and efficacy purposes.

The lack of publicly available data is not limited to fetal tissues PK. Values for some of the physiological parameters also had to be assumed. The flow rates which govern the solute transportation between the amniotic fluid and the fetus were measured in sheep [25,26]. As an initial assumption, those rates were used in the current PBPK model and scaled with body weight for fetus at earlier stages of pregnancy. Despite the assumption made above, the predicted fetal PK profiles are very reasonable in all simulated studies. The values for these rate constants may be revised in the future if more physiological information becomes available and/or based on simulations for additional APIs.

In addition to transport between the amniotic fluid and the fetus, transplacental transfer is a key element influencing drugs fetal exposure. For both CFX and CZ, placenta model structure had to be changed to permeability limited and observed fetal blood cord data was used to calibrate the permeability parameters driving the transfer of APIs between the maternal and fetal blood compartments. This structural change is justified as those APIs are substrates of MRP4. This transporter has been identified on the apical membrane of the placenta, limiting the transfer of its substrates into the fetal tissues [60,61]. However, a recent proteomic study could not quantify the amount of this transporter at different stages of pregnancy [62]. Therefore, the effect of MRP4 on CFX and CZ transplacental transfer was captured through the permeability parameters which lumped effect of MRP4 amount and activity in the placenta tissue. In both case studies, the model could reasonably simulate observed fetal blood cord data obtained during the second and third trimester of pregnancy. However, due to the placental structural changes and the lack of information about MRP4 placenta expression during the first trimester of pregnancy, PBPK model based extrapolation to predict CFX and CZ fetal transfer during this gestation period should be limited to an exploratory role and not dose adjustment decision making.

As this research project focused on renally cleared drugs, two APIs were identified for pregnancy PBPK model validation: CFX and CZ. In both cases, the API elimination pathway is a combination of passive renal filtration and active secretion mediated by transporters. Also, both drugs presented a linear PK across clinical doses which demonstrates the non-saturation of transporter mediated secretion in the kidney tubules. During pregnancy, both secretion and filtration of the maternal renal clearance are expected to change due to the physiological changes in GFR, kidney size, and plasma free fraction. The changes in GFR and free fraction are implemented in the model and the passive filtration was automatically scaled by the model. The active tubular secretion was incorporated by including the relevant transporters in the kidneys [33,39,40]. Although most of the transporters involved in CZ and CFX secretion in human had been identified with certainty and most of the Michaelis-Menten K_m_ parameters were based on experimental data. V_max_ values were fitted against clinical data. The current model assumes constant expression levels of the transporters per gram of kidney tissue and the increase in kidney size during pregnancy accounted for changes active tubular secretion. These assumptions resulted in accurate predictions of changes in renal clearance for pregnant women and accurate predictions in maternal PK and urinary secretion, for both test compounds.

In case of renally secreted drugs, the fetus does not contribute to the systemic clearance [17]. But fetal kidney secretion may play a role in API distribution in fetal tissues and the dynamics of the exchange with the amniotic fluid. As the simplified fetal PBPK model does not include a specific fetal kidney compartment, the effect of fetal kidney transporters could not be investigated. For CFX case study, the amniotic fluid concentrations time profiles were simulated fairly well but these concentrations were underpredicted for CZ. For both, the fetal renal clearance was estimated as fetal fup ∗ fetal GFR. It is possible that some of the transporters involve in CFX and CZ secretion have a partial activity during later stages of pregnancy. Ontogeny data for transporters in neonates, infants, and pediatrics population are also limited; therefore, the transporter expression levels could not be estimated by extrapolation from infant data as it has been done for other physiological parameters as described in “MATERIAL AND METHODS” section. At this stage, as the observed data were described accurately for CFX and within a reasonable range for CZ, no structural improvement was considered. The model will be refined in the future as the scientific knowledge evolves.

Prior to predicting the PK in maternal and fetal tissues, the PBPK model must be developed and validated against data in non-pregnant populations [52]. In this research project, the models for both compounds were first developed in healthy male subjects, validated in non-pregnant female subjects, and then applied to pregnant female subjects. Since the healthy volunteers are usually male subjects for the baseline models validation, when the same model is adopted and later applied to female subjects, there might be further adjustments need to be considered and an additional validation step may be necessary. For both CFX and CZ, postpartum data were available and used to refine the model if necessary. For CFX, OAT3 V_max_ had to be decreased by five-fold to match the amount of drug excreted in the urine for female healthy/postpartum subjects even if the plasma concentration was well predicted. For CZ case study, no adjustments were necessary to predict the postpartum dataset. Hence, it seems important to include the postpartum data whenever it is available to ensure an optimal quality by design modeling.

The number of subjects in pregnant clinical studies is usually limited, and the observed mean values for certain variables may be shifted from the default averaged population values, especially for drugs with highly variable pharmacokinetic parameters. Population simulations are a good way to access inter-subject variabilities. To test the pregnant PBPK model ability to predict the observed clinical variability for both the maternal plasma and fetal tissues, a population simulation was performed for CFX. With this method, the variability in maternal plasma concentration time course as well as for fetal tissues (venous return and amniotic fluid) could be described. Therefore, the population simulation has provided a possible way to access uncertainty and variations in the subjects, especially in the fetal subjects.

To conclude, the current model can describe reasonably well the API concentration in all fetal related compartments, including fetal blood and amniotic fluid, and also gave a very good prediction for the maternal concentrations. The future work will focus on adding a more detailed fetal PBPK model to assist prediction for local tissue concentrations.

## CONCLUSIONS

The work presented here has described the development and use of a PBPK model to predict the changes in PK during pregnancy for compounds mainly cleared renally. The current approach has provided reasonable predictions for both maternal and fetal exposure at different pregnancy stages, especially for the second and third trimester. Although this approach is promising, validation is still needed for the first trimester and will be performed if case studies are published. The inclusion of postpartum subject data to validate/fine-tune the model has proved to improve prediction for both maternal and fetal PK profiles. To conclude, the ability to probe into both the maternal and fetal concentrations have made the current model potentially very useful in a number of other areas including dose predictions, drug safety, pharmacodynamics, and drug-drug interactions.

## Supplementary Information

ESM 1 (DOCX 178 kb)

ESM 2 (DOCX 211 kb)

ESM 3 (DOCX 45 kb)

ESM 4 (DOCX 659 kb)

## References

[1] Mitchell AA, Gilboa SM, Werler MM, Kelley KE, Louik C, Hernández-Díaz S (2011). Medication use during pregnancy, with particular focus on prescription drugs: 1976–2008. Am J Obstet Gynecol.

[2] Lacroix I, Damase-Michel C, Lapeyre-Mestre M, Montastruc JL (2000). Prescription of drugs during pregnancy in France. Lancet.

[3] U.S. Food and Drug Administration Pregnant women: scientific and ethical considerations for inclusion in clinical trials guidance for industry [Internet]. 2018. Available from: https://www.fda.gov/media/112195/download

[4] European Medicines Agency. Guideline on risk assessment of medicinal products on human reproduction and lactation: from data to labelling [Internet]. 2009. Available from: https://www.ema.europa.eu/en/documents/scientific-guideline/guideline-risk-assessment-medicinal-products-human-reproduction-lactation-data-labelling_en.pdf

[5] Brent RL (2004). Utilization of animal studies to determine the effects and human risks of environmental toxicants (drugs, chemicals, and physical agents). Pediatrics..

[6] European Medicines Agency. Guideline on the exposure to medicinal products during pregnancy: need for post-authorisation data [Internet]. 2006. Available from: https://www.ema.europa.eu/en/documents/regulatory-procedural-guideline/guideline-exposure-medicinal-products-during-pregnancy-need-post-authorisation-data_en.pdf

[7] Vargesson N (2015). Thalidomide-induced teratogenesis: history and mechanisms. Birth Defects Res Part C Embryo Today Rev.

[8] Abduljalil K, Furness P, Johnson TN, Rostami-Hodjegan A, Soltani H (2012). Anatomical, physiological and metabolic changes with gestational age during normal pregnancy: a database for parameters required in physiologically based pharmacokinetic modelling. Clin Pharmacokinet.

[9] Abduljalil K, Johnson TN, Rostami-Hodjegan A (2018). Fetal physiologically-based pharmacokinetic models: systems information on fetal biometry and gross composition. Clin Pharmacokinet.

[10] Dallmann A, Ince I, Meyer M, Willmann S, Eissing T, Hempel G (2017). Gestation-specific changes in the anatomy and physiology of healthy pregnant women: an extended repository of model parameters for physiologically based pharmacokinetic modeling in pregnancy. Clin Pharmacokinet.

[11] Marsousi N, Desmeules JA, Rudaz S, Daali Y (2017). Usefulness of PBPK modeling in incorporation of clinical conditions in personalized medicine. J Pharm Sci.

[12] Luecke RH, Wosilait WD, Pearce BA, Young JF (1997). A computer model and program for xenobiotic disposition during pregnancy. Comput Methods Prog Biomed.

[13] De Sousa MM, Hirt D, Urien S, Valade E, Bouazza N, Foissac F (2015). Physiologically-based pharmacokinetic modeling of renally excreted antiretroviral drugs in pregnant women. Br J Clin Pharmacol.

[14] Ke AB, Nallani SC, Zhao P, Rostami-Hodjegan A, Isoherranen N, Unadkat JD (2013). A physiologically based pharmacokinetic model to predict disposition of CYP2D6 and CYP1A2 metabolized drugs in pregnant women. Drug Metab Dispos.

[15] Dallmann A, Ince I, Solodenko J, Meyer M, Willmann S, Eissing T, Hempel G (2017). Physiologically based pharmacokinetic modeling of renally cleared drugs in pregnant women. Clin Pharmacokinet.

[16] Xia B, Heimbach T, Gollen R, Nanavati C, He H (2013). A Simplified PBPK modeling approach for prediction of pharmacokinetics of four primarily renally excreted and CYP3A metabolized compounds during pregnancy. AAPS J.

[17] De Sousa MM, Lui G, Zheng Y, Pressiat C, Hirt D, Valade E (2017). A physiologically-based pharmacokinetic model to predict human fetal exposure for a drug metabolized by several CYP450 pathways. Clin Pharmacokinet.

[18] Zhang Z, Imperial MZ, Patilea-Vrana GI, Wedagedera J, Gaohua L, Unadkat JD (2017). Development of a novel maternal-fetal physiologically based pharmacokinetic model I: insights into factors that determine fetal drug exposure through simulations and sensitivity analyses. Drug Metab Dispos Biol Fate Chem.

[19] Zhang Z, Unadkat JD (2017). Development of a novel maternal-fetal physiologically based pharmacokinetic model II: verification of the model for passive placental permeability drugs. Drug Metab Dispos Biol Fate Chem.

[20] Carmichael S, Abrams B, Selvin S (1997). The pattern of maternal weight gain in women with good pregnancy outcomes. Am J Public Health.

[21] Institute of Medicine (US) and National Research Council (US) Committee to Reexamine IOM Pregnancy Weight Guidelines. Weight gain during pregnancy: reexamining the guidelines [Internet]. Rasmussen KM, Yaktine AL, editors. Washington (DC): National Academies Press (US); 2009 [cited 2020 Jul 28]. (The National Academies Collection: Reports funded by National Institutes of Health). Available from: http://www.ncbi.nlm.nih.gov/books/NBK32813/20669500

[22] Jopling J, Henry E, Wiedmeier SE, Christensen RD (2009). Reference ranges for hematocrit and blood hemoglobin concentration during the neonatal period: data from a multihospital health care system. Pediatrics..

[23] Rubin MI, Bruck E, Rapoport M, Snively M, McKay H, Baumler A (1949). Maturation of renal function in childhood: clearance studies. J Clin Invest.

[24] Yanowitz TD, Yao AC, Pettigrew KD, Werner JC, Oh W, Stonestreet BS (1999). Postnatal hemodynamic changes in very-low-birthweight infants. J Appl Physiol Bethesda Md 1985.

[25] Brace RA (1995). Progress toward understanding the regulation of amniotic fluid volume: water and solute fluxes in and through the fetal membranes. Placenta..

[26] Underwood M (2005). Amniotic fluid: not just fetal urine anymore. J Perinatol.

[27] Underwood MA, Gilbert WM, Sherman MP (2005). Amniotic fluid: not just fetal urine anymore. J Perinatol.

[28] Brace RA, Anderson DF, Cheung CY (2014). Regulation of amniotic fluid volume: mathematical model based on intramembranous transport mechanisms. Am J Phys Regul Integr Comp Phys.

[29] Lukacova V, Parrott NJ, Fraczkiewicz G, Bolger MB, Woltosz WS. General approach to calculation of tissue:plasma partition coefficients for physiologically based pharmacokinetic (PBPK) modeling. AAPS Annual Meeting; 2008; Atlanta.

[30] Poulin P, Theil FP (2000). A priori prediction of tissue:plasma partition coefficients of drugs to facilitate the use of physiologically-based pharmacokinetic models in drug discovery. J Pharm Sci.

[31] Verhagen CA, Mattie H, Van Strijen E (1994). The renal clearance of cefuroxime and ceftazidime and the effect of probenecid on their tubular excretion. Br J Clin Pharmacol.

[32] Sakurai Y, Motohashi H, Ueo H, Masuda S, Saito H, Okuda M, Mori N, Matsuura M, Doi T, Fukatsu A, Ogawa O, Inui KI (2004). Expression levels of renal organic anion transporters (OATs) and their correlation with anionic drug excretion in patients with renal diseases. Pharm Res.

[33] Ci L, Kusuhara H, Adachi M, Schuetz JD, Takeuchi K, Sugiyama Y (2007). Involvement of MRP4 (ABCC4) in the luminal efflux of ceftizoxime and cefazolin in the kidney. Mol Pharmacol.

[34] Foord RD (1976). Cefuroxime: human pharmacokinetics. Antimicrob Agents Chemother.

[35] Philipson A, Stiernstedt G (1982). Pharmacokinetics of cefuroxime in pregnancy. Am J Obstet Gynecol.

[36] Holt DE, Fisk NM, Spencer JA, de Louvois J, Hurley R, Harvey D (1993). Transplacental transfer of cefuroxime in uncomplicated pregnancies and those complicated by hydrops or changes in amniotic fluid volume. Arch Dis Child.

[37] Holt DE, Broadbent M, Spencer JA, de Louvois J, Hurley R, Harvey D (1994). The placental transfer of cefuroxime at parturition. Eur J Obstet Gynecol Reprod Biol.

[38] Oswald S, Müller J, Neugebauer U, Schröter R, Herrmann E, Pavenstädt H (2019). Protein abundance of clinically relevant drug transporters in the human kidneys. Int J Mol Sci.

[39] Ueo H, Motohashi H, Katsura T, Inui K (2005). Human organic anion transporter hOAT3 is a potent transporter of cephalosporin antibiotics, in comparison with hOAT1. Biochem Pharmacol.

[40] Uwai Y, Saito H, Inui K-I (2002). Rat renal organic anion transporter rOAT1 mediates transport of urinary-excreted cephalosporins, but not of biliary-excreted cefoperazone. Drug Metab Pharmacokinet.

[41] Smyth RD, Pfeffer M, Donald AG, Van Harken R, Hottendorf GH (1979). Clinical pharmacokinetics and safety of high doses of ceforanide (BL-S786R) and cefazolin. Antimicrob Agents Chemother.

[42] Singhvi SM, Heald AF, Schreiber EC (1978). Pharmacokinetics of cephalosporin antibiotics: protein-binding considerations. Chemotherapy..

[43] Rattie ES, Ravin LJ (1975). Pharmacokinetic interpretation of blood levels and urinary excretion data for cefazolin and cephalothin after intravenous and intramuscular administration in humans. Antimicrob Agents Chemother.

[44] Lee FH, Pfeffer M, Van Harken DR, Smyth RD, Hottendorf GH (1980). Comparative pharmacokinetics of ceforanide (BL-S786R) and cefazolin in laboratory animals and humans. Antimicrob Agents Chemother.

[45] Shimizu T (1974). Studies on protein binding of cefazolin and other antibiotics. Jpn J Antibiot.

[46] Philipson A, Stiernstedt G, Ehrnebo M (1987). Comparison of the pharmacokinetics of cephradine and cefazolin in pregnant and non-pregnant women. Clin Pharmacokinet.

[47] Elkomy MH, Sultan P, Drover DR, Epshtein E, Galinkin JL, Carvalho B (2014). Pharmacokinetics of prophylactic cefazolin in parturients undergoing cesarean delivery. Antimicrob Agents Chemother.

[48] Fiore Mitchell T, Pearlman MD, Chapman RL, Bhatt-Mehta V, Faix RG (2001). Maternal and transplacental pharmacokinetics of cefazolin. Obstet Gynecol.

[49] Brown CE, Christmas JT, Bawdon RE (1990). Placental transfer of cefazolin and piperacillin in pregnancies remote from term complicated by Rh isoimmunization. Am J Obstet Gynecol.

[50] Zhang Z, Imperial MZ, Patilea-Vrana GI, Wedagedera J, Gaohua L, Unadkat JD (2017). Development of a novel maternal-fetal physiologically based pharmacokinetic model I: insights into factors that determine fetal drug exposure through simulations and sensitivity analyses. Drug Metab Dispos.

[51] Zhang Z, Unadkat JD (2017). Development of a novel maternal-fetal physiologically based pharmacokinetic model II: verification of the model for passive placental permeability drugs. Drug Metab Dispos Biol Fate Chem.

[52] Abduljalil K, Badhan RKS (2020). Drug dosing during pregnancy-opportunities for physiologically based pharmacokinetic models. J Pharmacokinet Pharmacodyn.

[53] De Sousa MM, Hirt D, Vinot C, Valade E, Lui G, Pressiat C (2016). Prediction of human fetal pharmacokinetics using ex vivo human placenta perfusion studies and physiologically based models. Br J Clin Pharmacol.

[54] Dallmann A, Pfister M, van den Anker J, Eissing T (2018). Physiologically based pharmacokinetic modeling in pregnancy: a systematic review of published models. Clin Pharmacol Ther.

[55] Liu XI, Momper JD, Rakhmanina N, van den Anker JN, Green DJ, Burckart GJ (2020). Physiologically based pharmacokinetic models to predict maternal pharmacokinetics and fetal exposure to emtricitabine and acyclovir. J Clin Pharmacol.

[56] Chaphekar N, Caritis S, Venkataramanan R (2020). Model-informed dose optimization in pregnancy. J Clin Pharmacol.

[57] Song L, Yu Z, Xu Y, Li X, Liu X, Liu D, Zhou T (2020). Preliminary physiologically based pharmacokinetic modeling of renally cleared drugs in Chinese pregnant women. Biopharm Drug Dispos.

[58] Codaccioni M, Bois F, Brochot C (2019). Placental transfer of xenobiotics in pregnancy physiologically-based pharmacokinetic models: structure and data. Comput Toxicol.

[59] Codaccioni M, Brochot C (2020). Assessing the impacts on fetal dosimetry of the modelling of the placental transfers of xenobiotics in a pregnancy physiologically based pharmacokinetic model. Toxicol Appl Pharmacol.

[60] Walker N, Filis P, Soffientini U, Bellingham M, O’Shaughnessy PJ, Fowler PA (2017). Placental transporter localization and expression in the human: the importance of species, sex, and gestational age differences†. Biol Reprod.

[61] Dallmann A, Liu XI, Burckart GJ, van den Anker J (2019). Drug transporters expressed in the human placenta and models for studying maternal-fetal drug transfer. J Clin Pharmacol.

[62] Anoshchenko O, Prasad B, Neradugomma NK, Wang J, Mao Q, Unadkat JD (2020). Gestational age–dependent abundance of human placental transporters as determined by quantitative targeted proteomics. Drug Metab Dispos.

