# Supplementary material for: PBPK Modeling Approach to Predict the Behavior of Drugs Cleared by Kidney in Pregnant Subjects and Fetus
Source: AAPS J. 2021 Jun 24;23(4):89. doi: 10.1208/s12248-021-00603-y (PMC8225528; doi:10.1208/s12248-021-00603-y)
Supplement: Supplementary file 1 — (DOCX 178 kb) [file 12248_2021_603_MOESM1_ESM.docx]

*Supplementary Material 1: Model Equations:*

**Amniotic fluid** is a clear yellowish liquid that surrounds the fetus during pregnancy. This fluid is crucial in fetal development. It provides necessary nutrients such as electrolytes, liquids, carbohydrates, and proteins while serves as a barrier for bacteria growth and absorbs outside pressure and physical forces. Maintaining the normal amount of fluid ensures the fetus development. Conditions such as Oligohydramnios (too little fluid) or Polyhydramnios (too much fluid) may cause fetal growth disorder and preterm labor. The regulation of the fluid includes multiple pathways (1,2):

**Trans-membranous pathway**: transfer between amniotic fluid and uterus wall.

**Intra-membranous pathway**: interchange of the solute and water between amniotic fluid and fetal blood which occurs in placenta, fetal skin, and umbilical cord. This pathway can be split into active and passive components, where the former is driven by vesicular pressure and the latter depends on osmosity.

**Fetal pathway**: interchange of the solute and water between fetal tissue and amniotic fluid.

The above mentioned pathways are simplified and incorporated in the model. The rate constants for the movement of the water and solute are not well established for human. Some measurements are available for ovine fetus (1–3), which have been adopted and scaled by the fetal body weight. The homeostasis of amniotic fluid is described by the following equation:

$Urinary rate+K_{sec}=K_{sw}+K_{intram}$ Equation (1)

where $K_{sec}$ is the secretion rate from the fetal tissue, $K_{sw}$ is the fetal swallowing rate and $K_{intram}$ is the active part of intramembrouensous pathway.

The fetal tissue is modeled as one whole tissue in this model. The fetal pathways are modeled directly as interaction between the fetal tissue and the amniotic fluid (Equation 1-2). Depending on the tissue types, the interaction between the tissues will be modeled differently. For all perfusion tissues, we have the following equations:

$$V_{t}^{AF}\frac{dC_{t}^{AF}}{dt}=-K_{intraM\_osm}\left( C_{t,u}^{AF}-\frac{C_{t,u}^{Fetus}}{Kp_{Fetus}} \right)-K_{intraM\_osm}\left( C_{t,u}^{AF}-\frac{C_{t,u}^{FP}}{Kp_{FP}} \right)+(CL_{filt}*f_{u}^{Fetus}+Ksec)*\frac{C_{t}^{Fetus}}{Kp_{Fetus}}$$

$$-\left( K_{sw}+K_{intraM_{ves}} \right)*C_{t}^{AF}-Pstc_{transM}\left( C_{t,u}^{AF}-\frac{C_{t,u}^{Uterus}}{Kp_{Uterus}} \right)-K_{trans}\left( C_{t,u}^{AF}-\frac{C_{t,u}^{Fetus}}{Kp_{Fetus}} \right)-Clearance$$

Equation (2)

$$V_{t}^{Fetus}\frac{dC_{t}^{Fetus}}{dt}=Q_{Fetus}\left( Cb_{i}-\frac{C_{t}^{Fetus}*Rbp}{Kp_{Fetus}} \right)+K_{intraM\_osm}\left( C_{t,u}^{AF}-\frac{C_{t,u}^{Fetus}}{Kp_{Fetus}} \right)$$

$$-(CL_{filt}*f_{u}^{Fetus}+Ksec)*\frac{C_{t}^{Fetus}}{Kp_{Fetus}}+\left( K_{sw}+K_{intraM\_vas} \right)*C_{t}^{AF}-Clearance$$

Equation (3)

where ${K_{intraM\_osm}, K}_{intraM_{ves}}$ are intramembranous rate constants (defined in the amniotic fluid compartment), representing osmosis and vesicular routes, ${Pstc}_{trans}$ is the permeability between amniotic fluid and uterus, ${CL}_{filt}$ is the fetal filtration clearance, $C_{AF,u}$ is the unbound concentration in the amniotic fluid and $C_{Fetus}$ is the concentration in the fetal tissue. The solute exchange happens between the amniotic fluid and the vascular space of the fetal tissue. For permeability-limited fetal tissue, the fetal tissue concentration will be replaced by the fetal extravascular concentration in the above equations. In the equation, the $K_{intraM_{ves}}$ has set to be the same values as $K_{intram}$ in Equation 1, while $K_{intraM\_osm}$ is set to be 20% of $K_{intram}$ as suggested in (3).

The **placenta** tissue is modeled as two compartments: **maternal placenta** and **fetal placenta**, where the maternal placenta is facing the maternal blood circulation and the fetal placenta is facing the blood circulation from the fetal side. The equations for both tissue types are described below.

**Perfusion-limited placenta tissues:**

With perfusion-limited placenta (Figure 2), the redistribution of the drugs will be calculated instantaneously between the maternal and fetal placenta tissues at any given time.


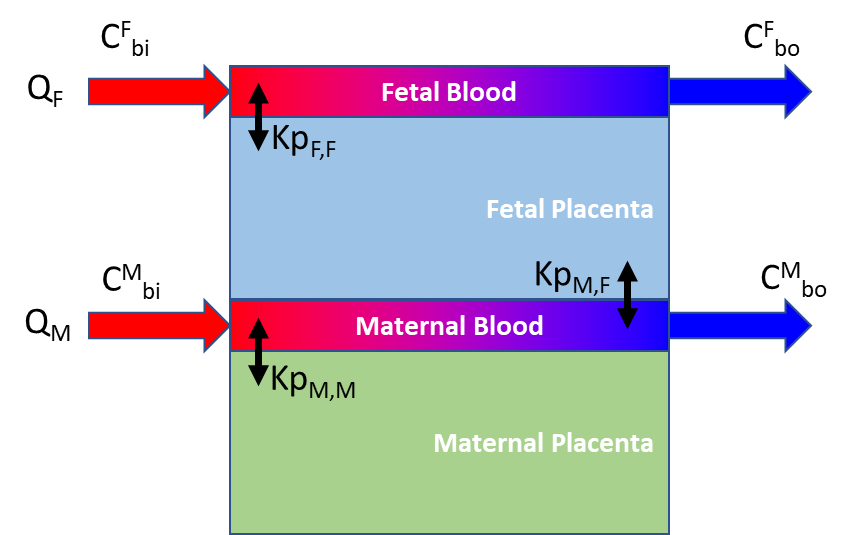


**Figure 2: Schematic of Perfusion-limited Placenta Tissues**

Total mass balance in the system is given by Equation 4 (without clearance terms, considering only instantaneous distribution parameterized by Kps):

$\frac{d(M_{t}^{M}+M_{t}^{F})}{dt}=Q_{M}\left(c_{bi}^{M}-c_{bo}^{M}\right)+Q_{F}\left(c_{bi}^{F}-c_{bo}^{F}\right)$ Equation (4)

With $c_{bo}^{M}=\frac{c_{t}^{Total}\times R_{bp}^{M}}{Kp_{T,M}}$and $c_{bo}^{F}=\frac{c_{t}^{F}\times R_{bp}^{F}}{Kp_{F,F}}$

where $M_{t}^{M},M_{t}^{F}$are the drug amounts (M means maternal placenta and F means Fetal placenta), $c_{bi}^{M},c_{bo}^{M}$ are the blood concentrations in (arterial) and out (venous) of maternal placenta, $c_{bi}^{F},c_{bo}^{F}$ are the blood concentrations in (arterial) and out (venous) of fetal placenta, $Q_{M},Q_{F}$are the blood flows in the placenta tissues, ${Kp}_{T,M}$ is the tissue/plasma partition coefficient between total placenta tissue (maternal and fetal) and maternal plasma, ${Kp}_{F,F}$ is the tissue/plasma partition coefficient between fetal tissue and fetal plasma.

Therefore, the **total concentration in placenta** is given by

$$c_{t}^{Total}=\frac{M_{t}^{M}+M_{t}^{F}}{V_{t}^{M}+V_{t}^{F}}=\frac{c_{t}^{M}V_{t}^{M}+c_{t}^{F}V_{t}^{F}}{V_{t}^{M}+V_{t}^{F}}=\frac{c_{t}^{F}\left(\frac{Kp_{M,M}}{Kp_{M,F}}V_{t}^{M}+V_{t}^{F}\right)}{V_{t}^{M}+V_{t}^{F}}=\frac{c_{t}^{M}\left(V_{t}^{M}+\frac{Kp_{M,F}}{Kp_{M,M}}V_{t}^{F}\right)}{V_{t}^{M}+V_{t}^{F}}$$

Equation (5)

where $V_{t}^{M},V_{t}^{F}$are the tissue volumes (M means maternal placenta and F means Fetal placenta), ${Kp}_{M,M}$ is the tissue/plasma partition coefficient between maternal tissue and maternal plasma, ${Kp}_{M,F}$ is the tissue/plasma partition coefficient fetal tissue and maternal plasma.

We can define:

$Kp_{T,M}=\frac{Kp_{M,M}V_{t}^{M}+Kp_{M,F}V_{t}^{F}}{V_{t}^{M}+V_{t}^{F}}$ Equation (6)

Substitute Equations 5 and 6 into equation 4 to obtain the general equation of change of drug amount in the placenta tissue (total maternal and fetal tissue):

$$\frac{d(M_{t}^{M}+M_{t}^{F})}{dt}=Q_{M}\left( c_{bi}^{M}-\frac{c_{t}^{Total}R_{bp}^{M}}{Kp_{T,M}} \right)+Q_{F}\left( c_{bi}^{F}-\frac{c_{t}^{F}R_{bp}^{F}}{Kp_{F,F}} \right)-clearance+transflux$$

With $transflux=K_{intraM\_osm}\left( C_{AF,u}-\frac{c_{t}^{F}}{Kp_{F,F}}*f_{u} \right)$ Equation (7)

where $R_{bp}^{M},R_{bp}^{F}$ are the blood to plasma concentration ratios in the maternal and fetal system.

For **fetal placenta**, we obtained:

$$\left(\frac{Kp_{M,M}}{Kp_{M,F}}V_{t}^{M}+V_{t}^{F}\right)\frac{d(c_{t}^{F})}{dt}=Q_{M}\left(c_{bi}^{M}-\frac{c_{t}^{F}\left(\frac{Kp_{M,M}}{Kp_{M,F}}V_{t}^{M}+V_{t}^{F}\right)R_{bp}^{M}}{Kp_{M,M}V_{t}^{M}+Kp_{M,F}V_{t}^{F}}\right)+Q_{F}\left(c_{bi}^{F}-\frac{c_{t}^{F}R_{bp}^{F}}{Kp_{F,F}}\right)-clearance+transflux$$

$$dM_{t}^{F}/dt=\frac{Kp_{M,F}*V_{t}^{F}}{Kp_{M,F}*V_{t}^{F}+Kp_{M,M}*V_{t}^{M}}\left[Q_{M}\left(C_{bi}^{M}-\frac{C_{t}^{F}}{Kp_{M,F}}R_{bp}^{M}\right)+Q_{F}\left(C_{bi}^{F}-\frac{C_{t}^{F}}{Kp_{F,F}}R_{bp}^{F}\right)-clearance+transflux\right]$$

Equation (8)

And for **maternal placenta**:

$$\left(V_{t}^{M}+\frac{Kp_{M,F}}{Kp_{M,M}}V_{t}^{F}\right)\frac{d(c_{t}^{M})}{dt}=Q_{M}\left(c_{bi}^{M}-\frac{c_{t}^{M}\left(V_{t}^{M}+\frac{Kp_{M,F}}{Kp_{M,M}}V_{t}^{F}\right)R_{bp}^{M}}{Kp_{M,M}V_{t}^{M}+Kp_{M,F}V_{t}^{F}}\right)+Q_{F}\left(c_{bi}^{F}-\frac{c_{t}^{F}R_{bp}^{F}}{Kp_{F,F}}\right)-clearance+transflux$$

Equation (9)

Fetal placenta tissue concentration may be further substituted to obtain final equation for maternal placenta:

$c_{t}^{F}=c_{t}^{M}\frac{Kp_{M,F}}{Kp_{M,M}}$ Equation (10)

$$\begin{matrix} dM_{t}^{M}/dt=\frac{Kp_{M,M}*V_{t}^{M}}{Kp_{M,F}*V_{t}^{F}+Kp_{M,M}*V_{t}^{M}}\times\\ \begin{matrix} \begin{matrix} \begin{matrix} & \end{matrix} & \end{matrix} & \end{matrix}\left[Q_{M}\left(C_{bi}^{M}-\frac{C_{t}^{M}}{Kp_{M,M}}R_{bp}^{M}\right)+Q_{F}\left(C_{bi}^{F}-\frac{C_{t}^{M}Kp_{M,F}}{Kp_{F,F}Kp_{M,M}}R_{bp}^{F}\right)-clearance+transflux\right] \end{matrix}$$

**Permeability-limited placenta tissues:**

When placenta is modeled as permeability-limited tissue, transplacental transfer will be enabled, which represents the interchange between the maternal blood plasma in the intervillous space and fetal trophoblastic cells (Figure 3). The surface area for the permeation is calculated from literature data (4,5) and is available for adjustment in the fetal placenta compartment.


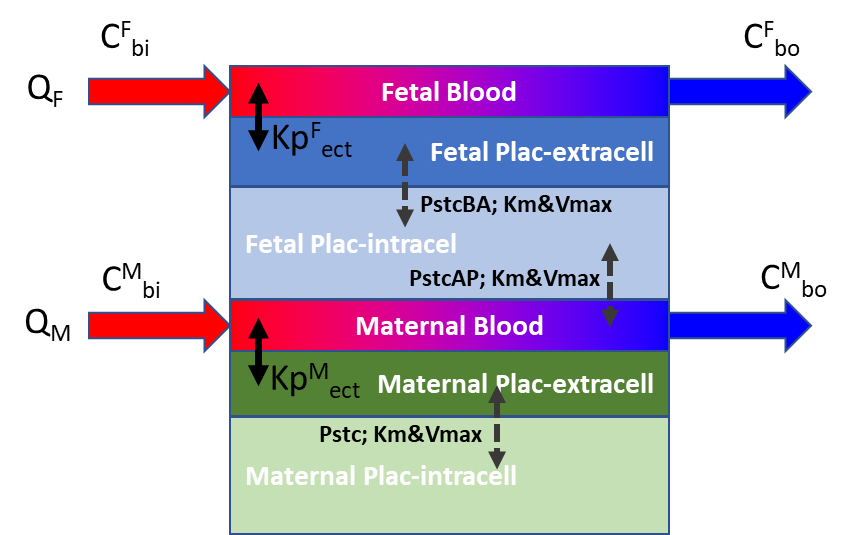


**Figure 3: Schematic of Permeability-limited Placenta Tissues**

For **Fetal Placenta**:

$permAP$and $permBA$ described the permeation between the fetal placenta tissue and maternal vascular space and the permeation between the tissue (fetal pleacenta or maternal placenta tissue) intracellular space and extracellular space accordingly, and $perm_{amniotic\_placenta}$ represents the drug permeation between the amniotic fluid and fetal placenta defined by the transmembraneous transport rate $K_{transM}$:

$$permAP=PstcAP\left(c_{p,u}^{M}-c_{ict,u}^{F}\right)+\nu_{\inf}c_{p,u}^{M}-\nu_{eff}c_{ict,u}^{F}$$

$$permBA=PstcBA\left(c_{ect,u}^{F}-c_{ict,u}^{F}\right)+\nu_{\inf}c_{ect,u}^{F}-\nu_{eff}c_{ict,u}^{F}$$

$$perm_{amniotic\_placenta}=K_{transM}\left(c_{AF,u}-c_{ect,u}^{F}\right)$$

$$V_{t}^{F}\frac{dc_{t}^{F}}{dt}=PermAP+PermBA-\nu_{metab}\left(c_{ict,u}^{F}\right)-CL_{t,u}^{F}c_{ict,u}^{F}$$

$\left( V_{ect}^{F}+V_{v}^{F}\frac{R_{bp}^{F}}{Kp_{ect}^{F}} \right)\frac{dc_{ect}^{F}}{dt}=Q_{F}\left( c_{bi}^{F}-\frac{c_{ect}^{F}R_{bp}^{F}}{Kp_{ect}^{F}} \right)-permBA+perm_{amniotic\_placenta}$

Equation (11)

For **Maternal placenta**:

$$permAP=PstcAP\left(c_{p,u}^{M}-c_{ict,u}^{F}\right)+\nu_{\inf}c_{p,u}^{M}-\nu_{eff}c_{ict,u}^{F}$$

$$permBA_{M}=PstcBA_{M}\left(c_{ect,u}^{M}-c_{ict,u}^{M}\right)+\nu_{\inf}c_{ect,u}^{M}-\nu_{eff}c_{ict,u}^{M}$$

$$V_{t}^{M}\frac{dc_{t}^{M}}{dt}=permBA_{M}-\nu_{metab}\left(c_{ict,u}^{M}\right)-CL_{t,u}^{M}c_{ict,u}^{M}$$

$$\left(V_{ect}^{M}+V_{v}^{M}\frac{R_{bp}^{M}}{Kp_{ect}^{M}}\right)\frac{dc_{ect}^{M}}{dt}=Q_{M}\left(c_{bi}^{M}-\frac{c_{ect}^{M}R_{bp}^{M}}{Kp_{ect}^{M}}\right)-permAP$$

Equation (12)

where $c_{ect,u}^{M}$ and $c_{ect,u}^{F}$ are drug concentrations in extracellular space (*u* = unbound) of maternal and fetal placentas, respectively; $c_{ict,u}^{M}$ and $c_{ict,u}^{F}$ are drug concentrations in intracellular space (*u* = unbound) of maternal and fetal placentas, respectively; $\nu_{metab}$ the metabolism rate in tissue, $\nu_{eff}$is the efflux transport rate from tissue, and$\nu_{\inf}$is the influx transport rate into tissue.

**References**

1. Brace RA. Progress toward understanding the regulation of amniotic fluid volume: Water and solute fluxes in and through the fetal membranes. Placenta. 1995 Jan 1;16(1):1–18.

2. Underwood MA, Gilbert WM, Sherman MP. Amniotic Fluid: Not Just Fetal Urine Anymore. J Perinatol. 2005 May;25(5):341–8.

3. Brace RA, Anderson DF, Cheung CY. Regulation of amniotic fluid volume: mathematical model based on intramembranous transport mechanisms. Am J Physiol Regul Integr Comp Physiol. 2014 Nov 15;307(10):R1260-1273.

4. Dallmann A, Ince I, Meyer M, Willmann S, Eissing T, Hempel G. Gestation-Specific Changes in the Anatomy and Physiology of Healthy Pregnant Women: An Extended Repository of Model Parameters for Physiologically Based Pharmacokinetic Modeling in Pregnancy. Clin Pharmacokinet. 2017;56(11):1303–30.

5. Zhang Z, Imperial MZ, Patilea-Vrana GI, Wedagedera J, Gaohua L, Unadkat JD. Development of a Novel Maternal-Fetal Physiologically Based Pharmacokinetic Model I: Insights into Factors that Determine Fetal Drug Exposure through Simulations and Sensitivity Analyses. Drug Metab Dispos Biol Fate Chem. 2017;45(8):920–38.
