# Supplementary material for: PBPK Modeling Approach to Predict the Behavior of Drugs Cleared by Kidney in Pregnant Subjects and Fetus
Source: AAPS J. 2021 Jun 24;23(4):89. doi: 10.1208/s12248-021-00603-y (PMC8225528; doi:10.1208/s12248-021-00603-y)
Supplement: Supplementary file 2 — (DOCX 211 kb) [file 12248_2021_603_MOESM2_ESM.docx]

*Supplementary Material 2: Model parameters*

**1.1 Weight gain**


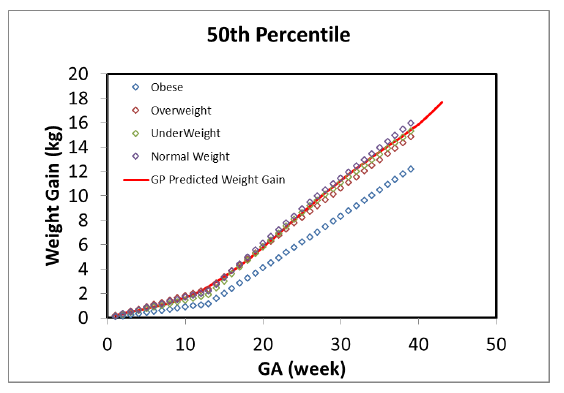


Figure 1: Maternal weight gain during pregnancy. Red solid line represents the calculation in GastroPlus, the color coded diamonds are the observed weight gain for obese, overweight, normal weight and underweight subjects (1). The equation fitted in the above figure is given as below:

Weight gain (kg) = 7.98E-07 GA^5^ - 8.41E-05 GA^4^ + 2.86E-03 GA^3^ - 2.65E-02 GA^2^ + 2.21E-01 GA

**1.2 Fetal weight and height**


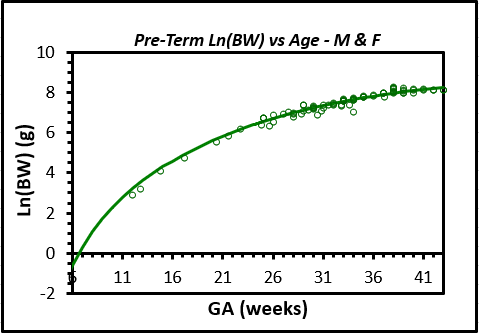

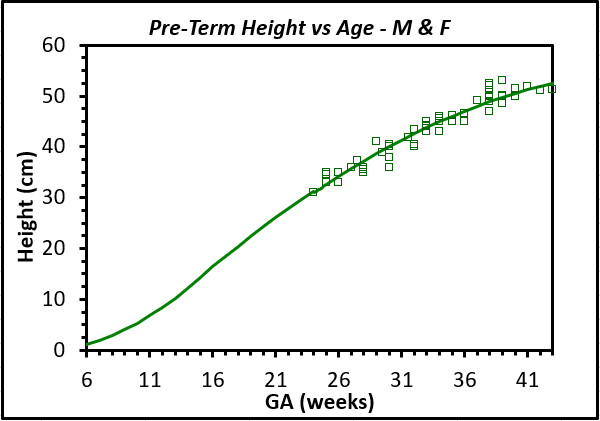


Figure 2: Fetal weight (left) and height (right) during pregnancy. These values were extrapolated from the preterm (12-16 weeks) infant and fetus measurements (2-8). Note that the calculated values before 6 weeks are not shown because only the uterus compartment will be included in the model. For the gestational age between 2 to 15 weeks, the equations from [34] were adopted because of lack of data. The equations in both figures are given as:

Weight (kg) = 1E-5 + (7.3472 * (GA- 2) ^ 4.23) / (38.26 ^ 4.23 + (GA - 2) ^ 4.23) (2$<$GA $\leq$ 15 weeks)

Weight (kg) = EXP (7.08E-9 * GA^5^ - 2.34E-6 * GA^4^ + 3.09E-4 * GA^3^ - 0.021 * GA^2^ + 0.76 * GA- 10.24) (GA > 15 weeks)

Height(cm) = 0.01 + (66.74 *(GA-2) ^2.32^) / (23^2.32^+ (GA-2) ^2.32^) (2$<$GA$\leq$15 weeks)

Height(cm) = -4.50E-7 * GA^4^ + 2.39E-4 * GA^3^ - 0.041 * GA^2^ + 3.22 * GA - 25.51 (GA > 15 weeks)

**1.3 Fetal hematocrit and GFR**


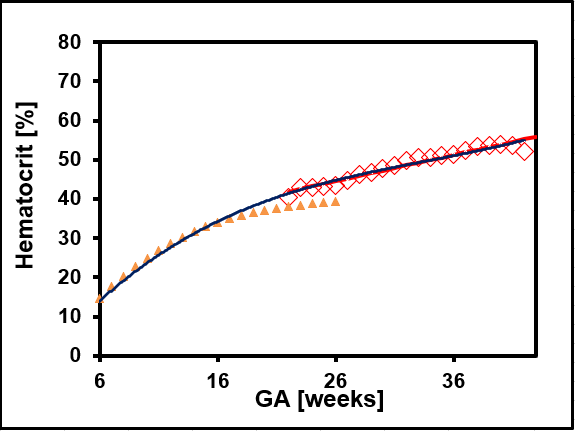


Figure 3: Plot of hematocrit vs GA for fetus or post-menstrual age (PMA) for infants. Individual colors represent data calculated by the equation provided in Dallmann (9) and literature fetus data (red) (10). The final hematocrit equation for fetus was fitted with the Dallmann equation and rest of the observed data (black line) and is given as below:

Hematocrit (%) = 9.17E-4 * GA^3^ - 0.0928 * GA^2^ + 3.713 * GA - 5.14


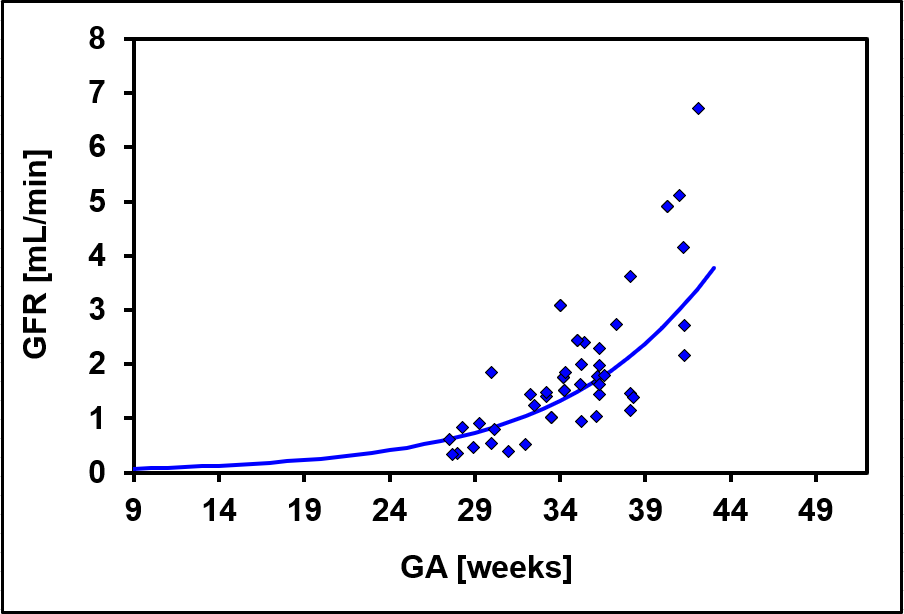


Figure 4: Plot of GFR vs GA for fetus or post-menstrual age (PMA) for infants. The earliest observed values were measured for preterm infants at 28 weeks. The measured data (blue diamond) were taken from (12-15) where data from infants less than 2 days old were used for this fitting. We have extrapolated the curve to calculate the GFR for fetus younger than 28 weeks old. The urine starts to form around 9 weeks hence the GFR has set to be 0 before 9 weeks old.

GFR =$0.02499*Exp\left( 0.1167* GA \right)$

- 1. **fetal plasma protein**

The total fetal plasma protein level is then calculated by the sum of albumin and alpha-1-acid glycoprotein.

**
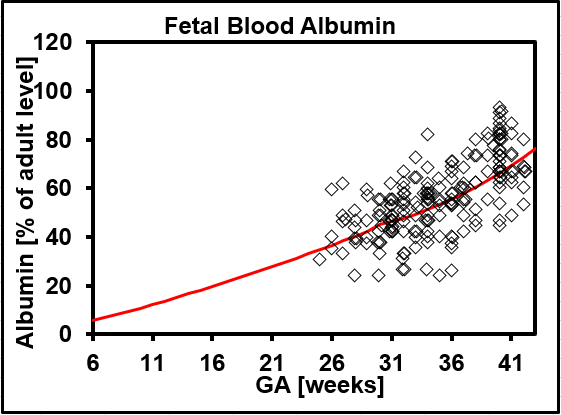
**

Figure 5: Plot of fetal blood albumin (as of adult level) vs GA. The earliest observed values were measured for preterm infants at 25 weeks (16-22). We have extrapolated the curve to calculate the albumin percent for fetus less than 25 weeks.

Alb (%) = $\left\{ \begin{matrix} 0.5675*{GA}^{1.2788} , GA<30 \\ 0.0915 *{GA}^{2} - 4.2769 * GA + 90.928 \end{matrix} \right.$

*
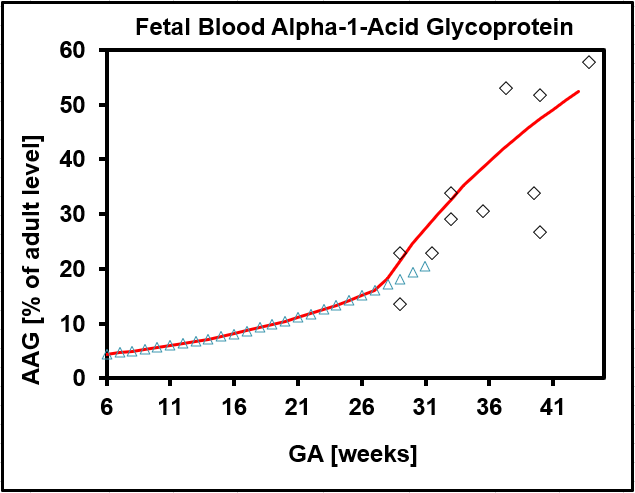
*

Figure 6: Plot of fetal blood AAG (as of adult level) vs GA. The earliest observed values (squares) were measured for preterm infants at 28 weeks (20, 23-24). The triangles represent the values calculated by Zhang’s equation as in (25). We have combined both sets of data to calculate the AAG percent over the entire range.

AAG (%) = $\left\{ \begin{matrix} 2 *\frac{EXP\left( 0.0616 *GA \right)}{0.657} , GA<28 \\ 1051 *\frac{GA}{(3.1 + GA)} - 928) \end{matrix} \right.$

The fetal plasma protein is then calculated as (0.657 * AAG + 45 * Alb) / (0.657 + 45) where 0.657 and 45 are the albumin and alpha-1 acid glycoprotein concentrations in healthy adults, and AAG and Alb are in the unit of percent of adult level as defined in the previous equations. The fraction unbound in the fetal plasma is then calculated as (McNamara, AAPS PharmSci, 2002, E4):

${fu}_{ped}=\frac{1}{1+\frac{P_{ped}}{P_{adult}}\frac{{1-fu}_{adult}}{{fu}_{adult}}}$,

where *P_ped_* and *P_adult_* is binding protein concentration in pediatric and adult subject, respectively; *fu_ped_* and *fu_adult_* is fraction unbound in plasma in pediatric and adult subject, respectively.

- 1. **Amniotic Fluid**

*
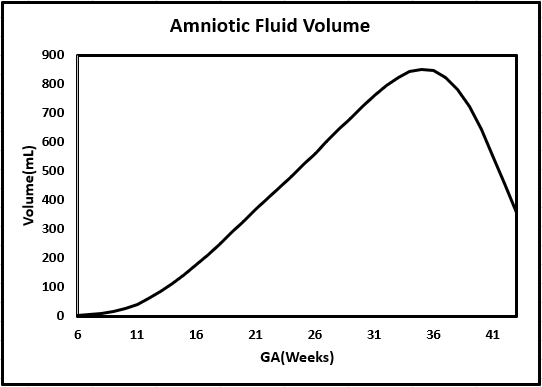
*

Figure 7: Plot of amniotic fluid vs GA for fetus. The amniotic fluid volume is calculated from the equation provided in (9), (see [9] supplemental material Table 1).

Volume (mL) = 1000 * ( -1.69E-5 * $\mathrm{FA}^{4}$ + 1.69E-3 * $\mathrm{FA}^{3}$- 0.0653 *$\mathrm{FA}^{2}$+1.22 *FA-10).

- 1. **Placenta Villous Area and Trans-placenta Transport**

The equation was adopted from [9] table 1 (FA means fertilization age):

Villous area = 20.3exp(−8.03exp(−0.0710*FA))−0.00659

The Volume of placenta was adopted from 1:

Placenta volume =0.937exp(−6.10exp(−0.0813FA))−0.00211

From [8], the average placenta surface area falls between 200-320 cm^2. We assume that the tissue surface area is 250 cm^2 at term (40 weeks). The fetal placenta tissue fraction was calculated from (1 - 0.525) from table 2 in [9]. Therefore, a scale factor of surface area enhancement at GA = 40 weeks is as below:

$Surface Enhancement Factor =\frac{villous area}{250*Fetal placenta Tissue Fraction}$.

The permeability surface area product (PStc) is then calculated as (PStc * Surface Enhancement Factor) to account for the villous surface area.

- 1. **fetal pathways values(30–32):**

The final rate constants are calculated as the value in the column of mL/day/kg multiplied by the fetal weight (section 1.2)

| Fetal rate constants | Flow rate at full term, L/day | Flow Rate, ml/day/kg |
| --- | --- | --- |
| Urinary rate | 1.26 | 380 |
| Lung secretion ($K_{sec}$) | 0.20 | 60 |
| Swallowing ($K_{sw}$) | 0.73 | 220 |
| Intramembraneous Pathway ($K_{intraM_{ves}})$ | 0.73 | 220 |
| Transmembraneous Pathway ($K_{transM})$ | 0.03 | 10 |

Tissue compositions:

The vascular fraction, interstitial fraction and intracellular fraction, volume fractions of proteins and lipids for fetal tissue, uterus and placenta tissue (maternal and fetal) were taken from Table 2 in reference [9]. Kp_(F,F), Kp_(M,M), Kp_(M,F) are then calculated by the Kp methods selected with the placenta tissue compositions and the fetal (the plasma protein change was given in 1.4) or maternal plasma compositions.

Organ blood flow rates：

Uterus, placenta, brain blood flow rates are taken from reference [9] table 2 and calculated as mL/min/mL tissue (the tissue volumes were also taken from the same table).

Other maternal changes:

The changes in other maternal physiological parameters at different gestational ages relative to values in nonpregnant subjects are described by equations from [9] and [32].

$$Albumin serum concentration change\left( \% \right)=100*\frac{14.7 Exp\left( -0.0454*FA \right)+31.7}{46.4}$$

$$AAG serum concentration change\left( \% \right)=100*\frac{7.68E-5*{FA}^{2}-0.00573*FA+0.701}{0.701}$$

$$Serum Hematocrit change\left( \% \right)=100*\frac{0.000401 * {FA}^{3}- 0.018 * {FA}^{2} + 0.0299 * FA + 40.1}{0.701}$$

$$GFR change\left( \% \right)=100*\frac{114 + 3.2367 * GA - 0.0572 * \mathrm{GA}^{2}}{114}$$

$$Plasma volume change\left( \% \right)=100*\frac{0.0000817*\mathrm{FA}^{3} + 0.00395 * \mathrm{FA}^{2} + 0.0000841*FA + 2.36}{2.36}$$

$Uterus volume \left( \mathrm{mL} \right)=\frac{0.959}{(1+28.9exp(-0.177*FA)}+0.048$

**References**

1. Carmichael S, Abrams B, Selvin S. The pattern of maternal weight gain in women with good pregnancy outcomes. Am J Public Health. 1997 Dec;87(12):1984–8.

2. Bertino, E., Coscia, A., et al. (2009). "Weight growth velocity of very low birth weight infants: role of gender, gestational age and major morbidities." Early Hum Dev 85(6): 339-47.

3. Chen, T.H. (1998). "A further discussion on fetal organ growth model." Chinese J Med Phys 15(4): 217-220.

4. Cooke, R.J. and Griffin, I. (2009). "Altered body composition in preterm infants at hospital discharge." Acta Paediatr 98(8): 1269-73.

5. Snyder, W.S., Cook, M.J., et al. (1975). "Report of the Task Group on reference man (ICRP Publication 23)." Elsevier Science Inc., pp.480

6. Usher, R. and McLean, F. (1969). "Intrauterine growth of live-born Caucasian infants at sea level: standards obtained from measurements in 7 dimensions of infants born between 25 and 44 weeks of gestation." J Pediatr 74(6): 901-10.

7. Wang, J. (1998). "Reference values of anatomical, physiological and metabolic parameters for Chinese." Atomic Energy Press: 7-58.

8. Zhang, Z.Y., Zhang, J.P., et al. (1994). "The analysis of liver weight, age and body weight in 189 fetuses." J Jining Medical College 17(3): 45.

9. Dallmann A, Ince I, Meyer M, Willmann S, Eissing T, Hempel G. Gestation-Specific Changes in the Anatomy and Physiology of Healthy Pregnant Women: An Extended Repository of Model Parameters for Physiologically Based Pharmacokinetic Modeling in Pregnancy. Clin Pharmacokinet. 2017;56(11):1303–30.

10. Jopling, J., Henry, E., et al. (2009). "Reference ranges for hematocrit and blood hemoglobin concentration during the neonatal period: data from a multihospital health care system." Pediatrics 123(2): e333-7.

11. Rubin, M.I., Bruck, E., et al. (1949). "Maturation of renal function in childhood; clearance studies." J Clin Invest 28(5 Pt 2): 1144-62.

12. Arant BS. Developmental patterns of renal functional maturation compared in the human neonate. J Pediatr. 1978 May;92(5):705–12.

13. Coulthard MG. Maturation of glomerular filtration in preterm and mature babies. Early Hum Dev. 1985 Sep;11(3–4):281–92.

14. DeWoskin RS, Thompson CM. Renal clearance parameters for PBPK model analysis of early lifestage differences in the disposition of environmental toxicants. Regul Toxicol Pharmacol RTP. 2008 Jun;51(1):66–86.

15. Fawer CL, Torrado A, Guignard JP. Maturation of renal function in full-term and premature neonates. Helv Paediatr Acta. 1979 Feb;34(1):11–21.

16. Cartlidge, P.H. and Rutter, N. (1986). "Serum albumin concentrations and oedema in the newborn." Arch Dis Child 61(7): 657-60.

17. Colón, A.R. (1990). "Textbook of Pediatric Hepatology." Year Book Medical Publishers, Inc., pp.353

18. Darrow, D.C. and Cary, M.K. (1933). "The serum albumin and globulin of newborn, premature and normal infants." J Pediatr 3: 573-9.

19. Ehrnebo, M., Aqurell, S., et al. (1971). "Age differences in drug binding by plasma proteins: studies on human foetuses, neonates and adults." Eur J Clin Pharmacol 3(4): 189-93.

20. Kanakoudi, F., Drossou, V., et al. (1995). "Serum concentrations of 10 acute-phase proteins in healthy term and preterm infants from birth to age 6 months." Clin Chem 41(4): 605-8.

21. Rane, A., Lunde, P.K., et al. (1971). "Plasma protein binding of diphenylhydantoin in normal and hyperbilirubinemic infants." J Pediatr 78(5): 877-82.

22. Reading, R.F., Ellis, R., et al. (1990). "Plasma albumin and total protein in preterm babies from birth to eight weeks." Early Hum Dev 22(2): 81-7.

23. Lerman, J., Strong, H.A., et al. (1989). "Effects of age on the serum concentration of alpha 1-acid glycoprotein and the binding of lidocaine in pediatric patients." Clin Pharmacol Ther 46(2): 219-25.

24. Philip, A.G. and Hewitt, J.R. (1983). "Alpha 1-acid glycoprotein in the neonate with and without infection." Biol Neonate 43(3-4): 118-24.

25. Zhang, Z., Imperial, M.Z., et al. (2017). "Development of a Novel Maternal-Fetal Physiologically Based Pharmacokinetic Model I: Insights into Factors that Determine Fetal Drug Exposure through Simulations and Sensitivity Analyses." Drug Metab Dispos 45(8): 920-938.

26. Chen, T.H. (1998). "A further discussion on fetal organ growth model." Chinese J Med Phys 15(4): 217-220.

27. Hao, C., Li, W., et al. (1993). "The weight and volume of fetal viscera." Chinese J Anat 16(1): 77-80.

28. Snyder, W.S., Cook, M.J., et al. (1975). "Report of the Task Group on reference man (ICRP Publication 23)." Elsevier Science Inc., pp.480

29. Ogiu, N., Nakamura, Y., et al. (1997). "A statistical analysis of the internal organ weights of normal Japanese people." Health Phys 72(3): 368-83.

30. Underwood MA, Gilbert WM, Sherman MP. Amniotic Fluid: Not Just Fetal Urine Anymore. J Perinatol. 2005 May;25(5):341–8.

31. Brace RA. Progress toward understanding the regulation of amniotic fluid volume: Water and solute fluxes in and through the fetal membranes. Placenta. 1995 Jan 1;16(1):1–18.

32. Abduljalil K, Furness P, Johnson TN, Rostami-Hodjegan A, Soltani H. Anatomical, physiological and metabolic changes with gestational age during normal pregnancy: a database for parameters required in physiologically based pharmacokinetic modelling. Clin Pharmacokinet. 2012 Jun 1;51(6):365–96.

33. Michael R. Syme, James W. Paxton and Jeffrey A. Keelan, “Drug Transfer and Metabolism by the Human Placenta”, Clin Pharmacokinet 43 no.8 (2004): 487-514

34. Abduljalil K, Johnson TN, Rostami-Hodjegan A. Fetal Physiologically-Based Pharmacokinetic Models: Systems Information on Fetal Biometry and Gross Composition. Clin Pharmacokinet. 2018;57(9):1149–71.
