# Supplementary material for: PBPK Modeling Approach to Predict the Behavior of Drugs Cleared by Kidney in Pregnant Subjects and Fetus
Source: AAPS J. 2021 Jun 24;23(4):89. doi: 10.1208/s12248-021-00603-y (PMC8225528; doi:10.1208/s12248-021-00603-y)
Supplement: Supplementary file 3 — (DOCX 45 kb) [file 12248_2021_603_MOESM3_ESM.docx]

*Supplementary Material 3: Model parameters and data origins:*

Table 1 summarizes the available clinical data used for model verification in healthy subject as well as clinical trials used for in silico predictions of pharmacokinetic in pregnant population:

Table 1: Clinical data used for the development and verification of models for CFX and CZ.

|  | **Dose (g)** | **Administration Route** | **Population** | **Source** |
| --- | --- | --- | --- | --- |
| **Cefuroxime** | | | | |
| *Baseline Model* | | | | |
|  | 0.25/0.5/1 | IV bolus | healthy | (1) |
| *Pregnancy model* | | | | |
|  | 0.75 | IV bolus | Post-partum | (2) |
|  | 0.75 | IV bolus | Pregnant | (2) |
|  | 0.75/1.5 | IV infusion | Pregnant | (3) |
|  | 0.75 | IV bolus | Pregnant | (4) |
| **Cefazolin** | | | | |
| *Baseline Model* | | | | |
|  | 1 | IV bolus | healthy | (5) |
|  | 1 | IV bolus | healthy | (6) |
|  | 2 | IV infusion | healthy | (7) |
|  | 2/3/4 | IV infusion | healthy | (8) |
| *Pregnancy model* | | | | |
|  | 0.5 | IV bolus | Post-partum | (9) |
|  | 0.5 | IV bolus | Pregnant | (9) |
|  | 1 | IV bolus | Pregnant | (10) |
|  | 1 | IV infusion | Pregnant | (11) |
|  | 2 | IV bolus | Pregnant | (12) |

For all compounds, the tissues included in the PBPK models are perfusion limited except for the kidney and placenta that are permeability limited in the final predictions. The drug specific parameters (e.g., Log P and pKa) used as input data and their references for CFX and CZ are summarized in Table 2 and Table 3.

Table 2: Key physicochemical and biopharmaceutical parameters for CFX used in GastroPlus simulations

| **Parameter** | **Value** | **Reference** |
| --- | --- | --- |
| *Physicochemical Properties* | | |
| logP | -0.16 | (13) |
| logD | 3.06 @ pH=6.5 | ADMET Predictor*^a^* |
| Diffusion coefficient | 0.69x10^-5^ cm^2^/s | ADMET Predictor*^a^* |
| S+pKa | 11.52 (Acid) | ADMET Predictor*^a^* |
|  | 10.24 (Acid) | ADMET Predictor*^a^* |
|  | 2.87 (Acid) | ADMET Predictor*^a^* |
| Native Aq. Solubility (S+Sw) | 4.29 mg/mL @ pH = 2.51 | ADMET Predictor |
| Blood:plasma concentration ratio (R_bp_) | 0.6 | Fitted (1) |
| Plasma protein binding (fup) | 67 % | (1) |
| Adjusted fup | 66.9 % | GastroPlus algorithm*^b^* |
| Specific Pstc | 0.2 mL/s | Fitted (2) |
| *Renal Clearance*  Filtration | fup*GFR^c^ | GastroPlus algorithm*^b^* |
| *Transporters* |  |  |
| MRP4 K_m_ | 248 μg/mL | assumed |
| MRP4 V_max_ | 0.0144/0.03 nmol/min/mg Prot. (0.02/0.004^d^ mg/s/mg transporters) | Fitted (1) |
| OAT3 K_m_ | 248 μg/mL | (14) |
| OAT3 V_max_ | 0.172 nmol/min/mg Prot. (0.23 mg/s/mg transporters) | Fitted (1) |
| *^a^* Predicted using ADMET Predictor v9.5  *^b^* Adjusted Fup was calculated from experimental Fup and S+logD @ pH = 7.4 using the default GastroPlus equation  *^c^* for both maternal and fetal kidney clearance  *^d^* 0.02 in healthy male subjects, 0.004 in pregnant and post-partum female subject | | |

Table 3: Key physicochemical and biopharmaceutical parameters for CZ used in GastroPlus simulations

| **Parameter** | **Value** | **Reference** |
| --- | --- | --- |
| *Physicochemical Properties* | | |
| logP | -0.24 | (13) |
| logD | 3.06 @ pH=6.5 | ADMET Predictor*^a^* |
| Diffusion coefficient | 0.68x10^-5^ cm^2^/s | ADMET Predictor*^a^* |
| pKa | 2.1 (Acid) | (15) |
| Reference solubility | 350 mg/mL @ pH = 7 | (16) |
| Blood:plasma concentration ratio (R_bp_) | 0.69 | ADMET Predictor*^a^* |
| Plasma protein binding (fup) | 11 % | (8) |
| Adjusted fup | 11 % | GastroPlus algorithm*^b^* |
| Specific Pstc | 1 mL/s | Fitted (10) |
| *Renal Clearance*  Filtration | Fup*GFR^c^ | GastroPlus algorithm*^b^* |
| MRP4 Vmax | 0.0225 nmol/min/mg Prot.  (0.033 mg/s/mg transporters) | Fitted (5) |
| MRP4 Km | 36.8 μg/mL | (17) |
| OAT3 Vmax | 0.136 nmol/min/mg Prot.  (0.2 mg/s/mg transporters) | Fitted (5) |
| OAT3 Km | 53 μg/mL | (18) |
| *^a^* Predicted using ADMET Predictor v9.5  *^b^* Adjusted Fup was calculated from experimental Fup and logD @ pH = 7.4 using the default GastroPlus equation  *^c^* for both maternal and fetal kidney clearance | | |

**References**

1. Foord RD. Cefuroxime: Human Pharmacokinetics. Antimicrob Agents Chemother. 1976 May;9(5):741–7.

2. Philipson A, Stiernstedt G. Pharmacokinetics of cefuroxime in pregnancy. Am J Obstet Gynecol. 1982 Apr 1;142(7):823–8.

3. Holt DE, Broadbent M, Spencer JA, de Louvois J, Hurley R, Harvey D. The placental transfer of cefuroxime at parturition. Eur J Obstet Gynecol Reprod Biol. 1994 May 18;54(3):177–80.

4. Holt DE, Fisk NM, Spencer JA, de Louvois J, Hurley R, Harvey D. Transplacental transfer of cefuroxime in uncomplicated pregnancies and those complicated by hydrops or changes in amniotic fluid volume. Arch Dis Child. 1993 Jan;68(1 Spec No):54–7.

5. Rattie ES, Ravin LJ. Pharmacokinetic Interpretation of Blood Levels and Urinary Excretion Data for Cefazolin and Cephalothin After Intravenous and Intramuscular Administration in Humans. Antimicrob Agents Chemother. 1975 May;7(5):606–13.

6. Singhvi SM, Heald AF, Schreiber EC. Pharmacokinetics of cephalosporin antibiotics: protein-binding considerations. Chemotherapy. 1978;24(3):121–33.

7. Lee FH, Pfeffer M, Van Harken DR, Smyth RD, Hottendorf GH. Comparative Pharmacokinetics of Ceforanide (BL-S786R) and Cefazolin in Laboratory Animals and Humans. Antimicrob Agents Chemother. 1980 Feb;17(2):188–92.

8. Smyth RD, Pfeffer M, Donald AG, Van Harken R, Hottendorf GH. Clinical Pharmacokinetics and Safety of High Doses of Ceforanide (BL-S786R) and Cefazolin. Antimicrob Agents Chemother. 1979 Nov;16(5):615–21.

9. Philipson A, Stiernstedt G, Ehrnebo M. Comparison of the pharmacokinetics of cephradine and cefazolin in pregnant and non-pregnant women. Clin Pharmacokinet. 1987 Feb;12(2):136–44.

10. Elkomy MH, Sultan P, Drover DR, Epshtein E, Galinkin JL, Carvalho B. Pharmacokinetics of prophylactic cefazolin in parturients undergoing cesarean delivery. Antimicrob Agents Chemother. 2014 Jun;58(6):3504–13.

11. Fiore Mitchell T, Pearlman MD, Chapman RL, Bhatt-Mehta V, Faix RG. Maternal and transplacental pharmacokinetics of cefazolin. Obstet Gynecol. 2001 Dec;98(6):1075–9.

12. Brown CE, Christmas JT, Bawdon RE. Placental transfer of cefazolin and piperacillin in pregnancies remote from term complicated by Rh isoimmunization. Am J Obstet Gynecol. 1990 Sep;163(3):938–43.

13. Yoshimura F, Nikaido H. Diffusion of beta-lactam antibiotics through the porin channels of Escherichia coli K-12. Antimicrob Agents Chemother. 1985 Jan 1;27(1):84–92.

14. Verhagen CA, Mattie H, Van Strijen E. The renal clearance of cefuroxime and ceftazidime and the effect of probenecid on their tubular excretion. Br J Clin Pharmacol. 1994 Feb;37(2):193–7.

15. Dallmann A, Ince I, Solodenko J, Meyer M, Willmann S, Eissing T, et al. Physiologically Based Pharmacokinetic Modeling of Renally Cleared Drugs in Pregnant Women. Clin Pharmacokinet. 2017 Dec;56(12):1525–41.

16. Dallmann A, Ince I, Solodenko J, Meyer M, Willmann S, Eissing T, et al. Physiologically Based Pharmacokinetic Modeling of Renally Cleared Drugs in Pregnant Women. Clin Pharmacokinet. 2017;56(12):1525–41.

17. Ci L, Kusuhara H, Adachi M, Schuetz JD, Takeuchi K, Sugiyama Y. Involvement of MRP4 (ABCC4) in the luminal efflux of ceftizoxime and cefazolin in the kidney. Mol Pharmacol. 2007 Jun;71(6):1591–7.

18. Sakurai Y, Motohashi H, Ueo H, Masuda S, Saito H, Okuda M, et al. Expression levels of renal organic anion transporters (OATs) and their correlation with anionic drug excretion in patients with renal diseases. Pharm Res. 2004 Jan;21(1):61–7.
