# Supplementary material for: PBPK Modeling Approach to Predict the Behavior of Drugs Cleared by Kidney in Pregnant Subjects and Fetus
Source: AAPS J. 2021 Jun 24;23(4):89. doi: 10.1208/s12248-021-00603-y (PMC8225528; doi:10.1208/s12248-021-00603-y)
Supplement: Supplementary file 4 — (DOCX 659 kb) [file 12248_2021_603_MOESM4_ESM.docx]

*Supplementary Material 4: Additional simulations:*

**Cefuroxime:**


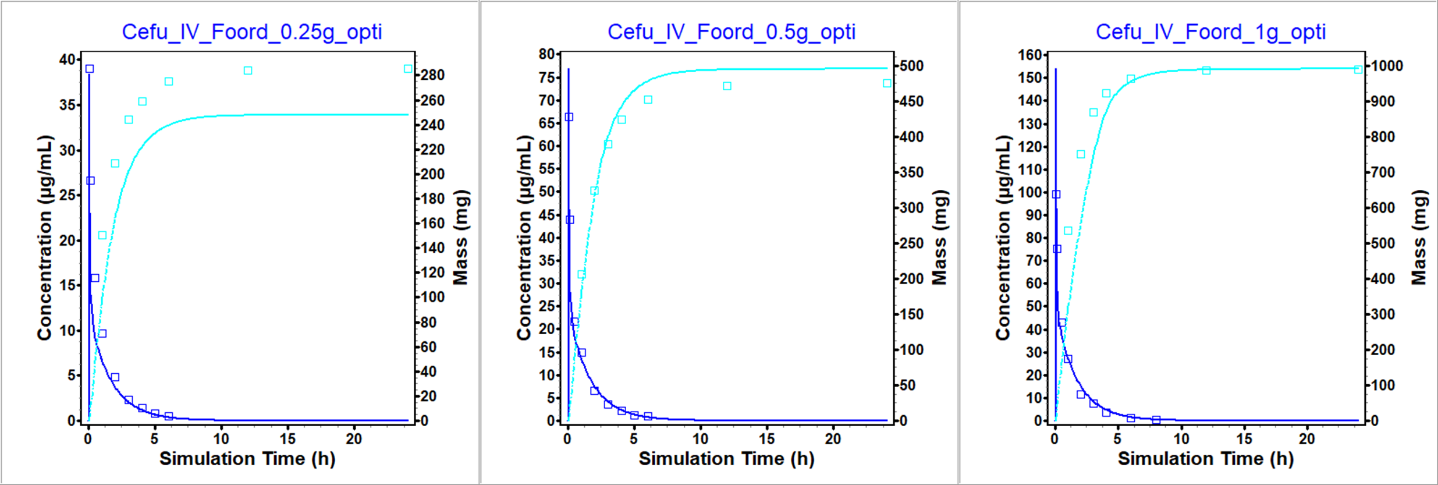


Figure 1: Simulated and observed (1) profiles after administration of CFX in healthy fasted volunteers (30-years-old, 70kg male subjects). Left. 0.25 g IV infusion. Middle. 0.5 g IV infusion Right. 1 g IV infusion. Blue lines and points represent plasma concentrations shown on the left y-axis. Light blue line represents percent of the dose excreted in urine shown on right y-axis.


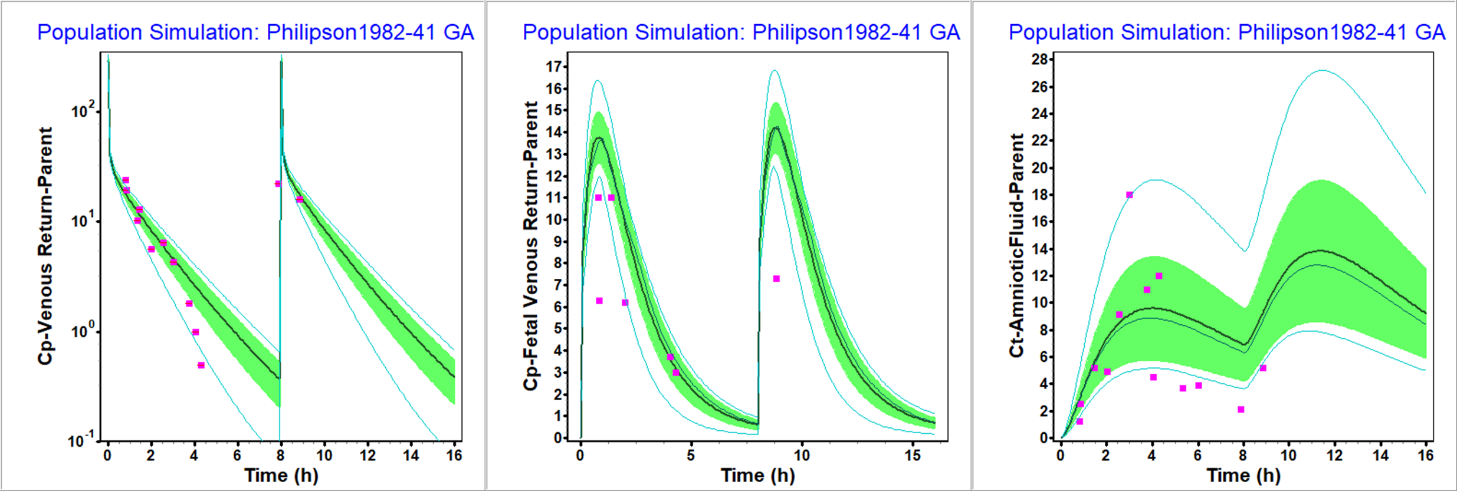


Figure 2: Population Simulation and observed PK profiles after administration of 0.75 g IV bolus CFX. The population setting used in the simulation matched the information provided in the original paper (7 subjects in total) (4). The CV% for the Vmax values for the two transporters are assumed to be 100%. The shaded green areas are the 90% confidence intervals while the two blue curves are the 100% probability contours.

**Cefazolin:**


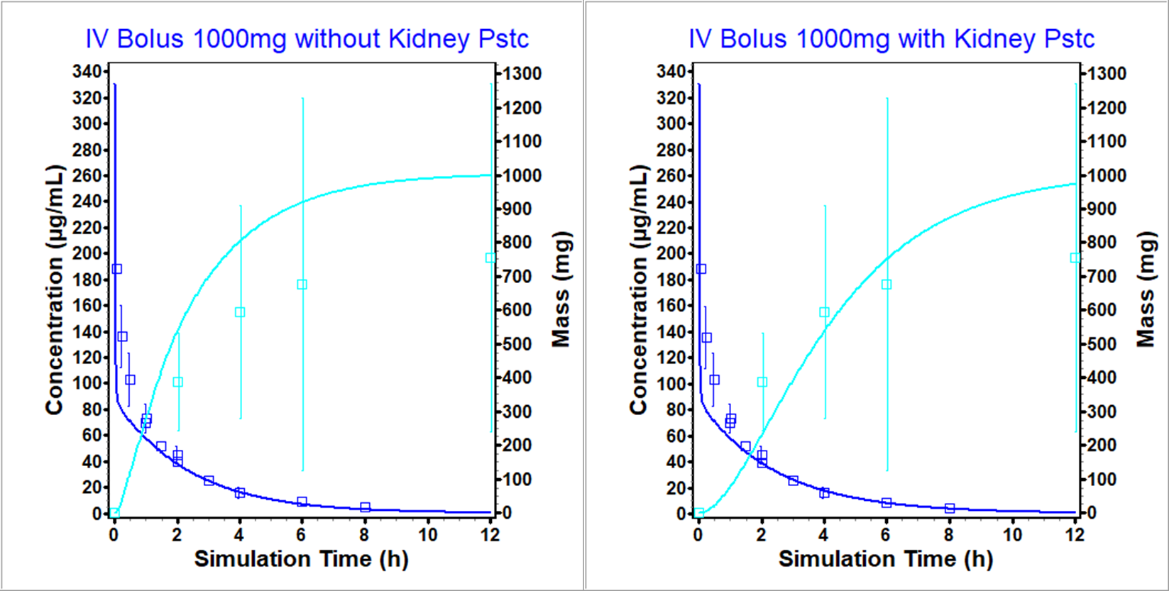


Figure 3: Simulated and observed (2,3) profiles after IV bolus 1g administration of CZ in healthy fasted volunteers (32-years-old, 79kg male subjects). Blue lines and points represent plasma concentrations shown on the left y-axis. Light blue line represents percent of the dose excreted in urine shown on right y-axis. Left plot does not include a kidney PStc whereas the simulation presented in the right pot include the kidney PStc (125.85 mL/s).

**References**

1. Foord RD. Cefuroxime: Human Pharmacokinetics. Antimicrob Agents Chemother. 1976 May;9(5):741–7.

2. Rattie ES, Ravin LJ. Pharmacokinetic Interpretation of Blood Levels and Urinary Excretion Data for Cefazolin and Cephalothin After Intravenous and Intramuscular Administration in Humans. Antimicrob Agents Chemother. 1975 May;7(5):606–13.

3. Singhvi SM, Heald AF, Schreiber EC. Pharmacokinetics of cephalosporin antibiotics: protein-binding considerations. Chemotherapy. 1978;24(3):121–33.

4. Philipson A, Stiernstedt G. Pharmacokinetics of cefuroxime in pregnancy. Am J Obstet Gynecol. 1982 Apr 1;142(7):823–8.
